# Exploring the targetome of IsrR, an iron-regulated sRNA controlling the synthesis of iron-containing proteins in *Staphylococcus aureus*

**DOI:** 10.3389/fmicb.2024.1439352

**Published:** 2024-07-05

**Authors:** Alexander Ganske, Larissa Milena Busch, Christian Hentschker, Alexander Reder, Stephan Michalik, Kristin Surmann, Uwe Völker, Ulrike Mäder

**Affiliations:** Interfaculty Institute for Genetics and Functional Genomics, University Medicine Greifswald, Greifswald, Germany

**Keywords:** *Staphylococcus aureus*, IsrR, sRNA, iron regulation, oxidative stress, proteome

## Abstract

*Staphylococcus aureus* is a common colonizer of the skin and nares of healthy individuals, but also a major cause of severe human infections. During interaction with the host, pathogenic bacteria must adapt to a variety of adverse conditions including nutrient deprivation. In particular, they encounter severe iron limitation in the mammalian host through iron sequestration by haptoglobin and iron-binding proteins, a phenomenon called “nutritional immunity.” In most bacteria, including *S. aureus*, the ferric uptake regulator (Fur) is the key regulator of iron homeostasis, which primarily acts as a transcriptional repressor of genes encoding iron acquisition systems. Moreover, Fur can control the expression of trans-acting small regulatory RNAs that play an important role in the cellular iron-sparing response involving major changes in cellular metabolism under iron-limiting conditions. In *S. aureus*, the sRNA IsrR is controlled by Fur, and most of its predicted targets are iron-containing proteins and other proteins related to iron metabolism and iron-dependent pathways. To characterize the IsrR targetome on a genome-wide scale, we combined proteomics-based identification of potential IsrR targets using *S. aureus* strains either lacking or constitutively expressing IsrR with an *in silico* target prediction approach, thereby suggesting 21 IsrR targets, of which 19 were negatively affected by IsrR based on the observed protein patterns. These included several Fe-S cluster- and heme-containing proteins, such as TCA cycle enzymes and catalase encoded by *katA*. IsrR affects multiple metabolic pathways connected to the TCA cycle as well as the oxidative stress response of *S. aureus* and links the iron limitation response to metabolic remodeling. In contrast to the majority of target mRNAs, the IsrR-*katA* mRNA interaction is predicted upstream of the ribosome binding site, and further experiments including mRNA half-life measurements demonstrated that IsrR, in addition to inhibiting translation initiation, can downregulate target protein levels by affecting mRNA stability.

## Introduction

As integral parts of gene regulatory networks, small regulatory RNAs (sRNAs) play important roles in the adaptation of bacteria to the respective environmental conditions. sRNAs are relatively short RNA molecules—typically 50 to 500 nucleotides in length—that act primarily as post-transcriptional regulators of gene expression, exerting their function by base-pairing with mRNAs or modifying the activity of regulatory proteins (reviewed in, e.g., Wagner and Romby, 2015). The former class of sRNAs, referred to as *trans*-acting sRNAs, usually regulate multiple mRNA targets by short, imperfect base pairing, which leads to changes in translation and/or mRNA stability. In bacterial pathogens, sRNAs are key elements in the regulation of virulence gene expression (Felden and Augagneur, 2021). That link has been first recognized with the discovery of the regulatory function of RNAIII of the Gram-positive bacterium *Staphylococcus aureus* (Novick et al., 1993), which is a common colonizer of the skin and nares of healthy individuals, but also a major cause of severe human diseases such as endocarditis and sepsis (Wertheim et al., 2005). During the past two decades, transcriptome studies using DNA tiling arrays or RNA-seq identified numerous putative sRNAs in *S. aureus*, of which less than 20 have been functionally characterized, revealing their association with various physiological processes, including virulence, metabolism, and antibiotic resistance (Menard et al., 2022). In contrast to protein-coding genes, homologs of most sRNAs are restricted to closely related bacteria (Updegrove et al., 2015); however, some sRNAs are more broadly conserved as, for example, staphylococcal RsaE (also named RoxS), an sRNA involved in the regulation of central metabolism, which is present in bacteria of the Bacillales order (Marincola et al., 2019). In addition, sRNAs can exhibit functional conservation without sharing sequence similarity, as demonstrated by a group of sRNAs expressed in response to iron limitation (Updegrove et al., 2015), which are found in Gram-negative and Gram-positive bacteria and comprise, for example, RyhB of *Escherichia coli* and FsrA of *Bacillus subtilis* (Oglesby-Sherrouse and Murphy, 2013).

Iron is an essential nutrient for virtually all organisms. It is involved as a cofactor in numerous metabolic processes, including the tricarboxylic acid (TCA) cycle, respiration, and DNA biosynthesis (Andrews et al., 2003). However, iron is also toxic to cells when present in excess because it promotes generation of hydroxyl radicals through the Fenton reaction, resulting in damage of cellular components and finally cell death (Imlay, 2013). Therefore, bacteria tightly control iron uptake, storage and utilization, and regulation of iron homeostasis is linked to the oxidative stress response, central metabolism and other cellular processes (Andrews et al., 2003). In their natural habitats, bacteria often have to cope with limited availability of iron, in particular due to the rapid oxidation of Fe^2+^ to Fe^3+^ under aerobic conditions and the low solubility of Fe^3+^ at neutral pH (Guerinot, 1994); hence, most bacteria rely on low molecular weight iron chelators, siderophores, for iron acquisition (reviewed in, e.g., Miethke and Marahiel, 2007). In the mammalian host, access to iron and other trace metals is particularly restricted in a process termed *nutritional immunity* by Weinberg (1975) (reviewed in, e.g., Jordan et al., 2020; Murdoch and Skaar, 2022). Most of the iron is complexed to heme, primarily found in hemoglobin that is captured by haptoglobin following hemolysis. Furthermore, extracellular free iron is sequestered within iron-binding proteins such as transferrin and lactoferrin. To proliferate under conditions of severe iron limitation, pathogenic bacteria require effective adaptation mechanisms, which especially comprise dedicated iron acquisition strategies (Cassat and Skaar, 2013; Palmer and Skaar, 2016; Sheldon et al., 2016). These strategies have been extensively studied in *S. aureus* that uses two general pathways for iron acquisition, heme uptake and siderophore-mediated iron uptake (reviewed in, e.g., Hammer and Skaar, 2011; Conroy et al., 2019).

The ferric uptake regulator (Fur), which senses intracellular iron levels, is the master regulator of iron homeostasis in Gram-negative and low G + C Gram-positive bacteria (Fillat, 2014). One important group of genes repressed by Fur under iron-sufficient conditions is involved in siderophore biosynthesis and iron uptake (reviewed in, e.g., Troxell and Hassan, 2013). When iron is limited, Fur dissociates from its target operators resulting in the induction of these systems. To adapt to iron limitation, bacteria also activate an “iron-sparing” response, allowing them to reduce the synthesis of iron-utilizing proteins that are not essential for growth. In different bacterial species, Fur indirectly controls the iron-sparing response by repressing the expression of an sRNA, which in turn inhibits the translation of mRNAs encoding non-essential iron-containing proteins. The first member of the aforementioned group of functionally analogous sRNAs, RyhB of *E. coli*, was identified by Massé and Gottesman (2002).

Based on several studies, as reviewed by Chareyre and Mandin (2018), the RyhB targetome of *E. coli* comprises more than 50 experimentally confirmed or very likely targets, which include genes encoding the TCA cycle enzymes aconitase and succinate dehydrogenase, iron storage proteins, and iron–sulfur (Fe-S) cluster assembly proteins, as well as genes involved in siderophore biosynthesis (reviewed in, e.g., Salvail and Massé, 2012). Whereas RyhB orthologs have been described in several members of Enterobacteriaceae and related species, functional analogous sRNAs are found all over the bacterial domain (Chareyre and Mandin, 2018), including Gram-positive bacteria where FsrA of *B. subtilis* is best characterized (Gaballa et al., 2008). FsrA negatively regulates, among others, the levels of aconitase (encoded by *citB*), succinate dehydrogenase (encoded by *sdhCAB*), glutamate synthase (encoded by *gltAB*), C4-dicarboxylate permease DctP, and the lactate utilization enzymes LutABC, thereby accounting for major changes in cellular metabolism under iron-limiting growth conditions (Smaldone et al., 2012a, 2012b).

**Figure 1:**
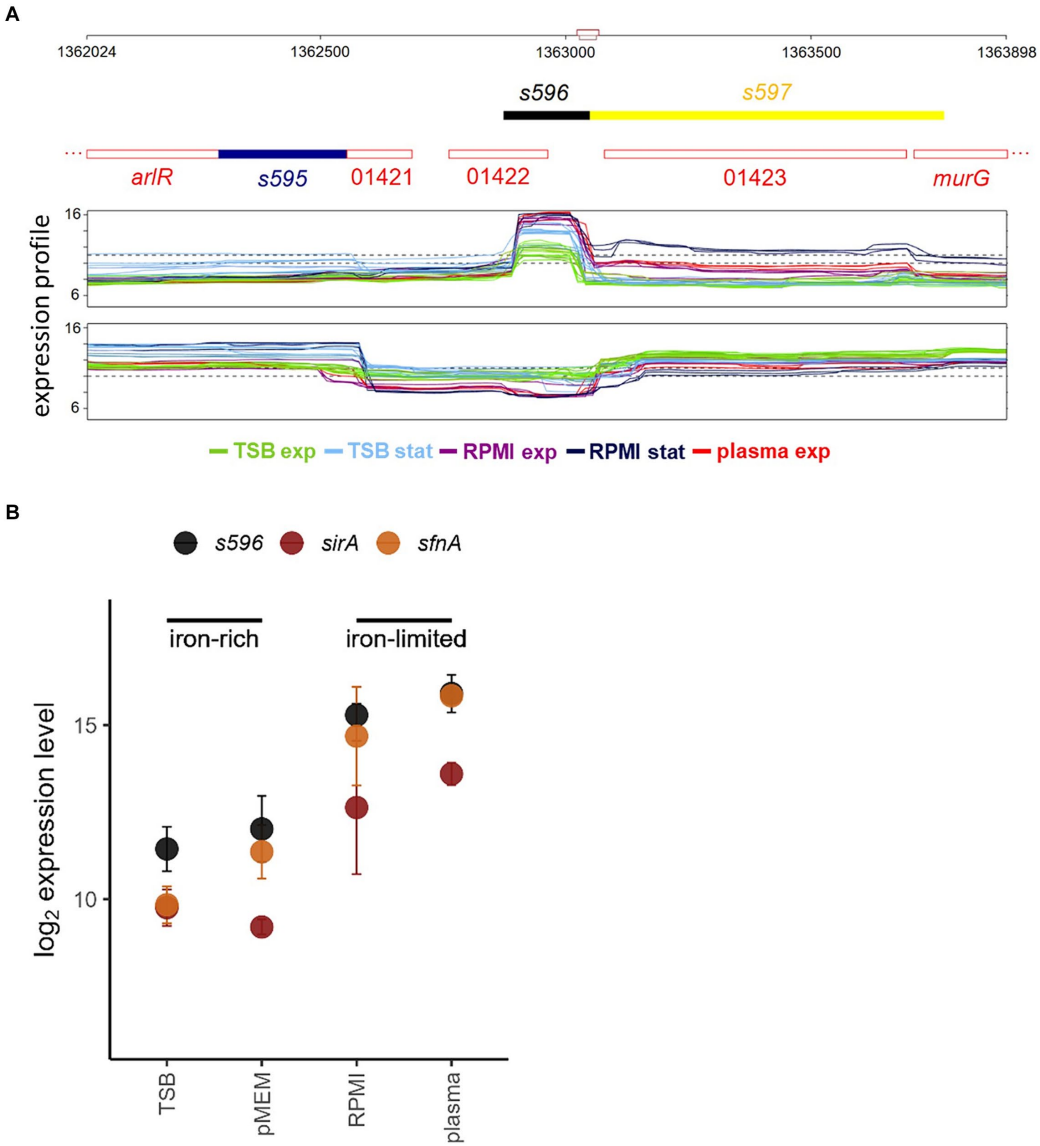
**(A)** Region of the *S. aureus* chromosome covering sRNA S596 as annotated by Mäder et al. (2016) visualized with Genoscapist (Dérozier et al., 2021). The upper track shows for both strands the CDS annotation from NCBI GenBank CP000253.1 (red numbers stand for the respective locus tags (SAOUHSC_01421, SAOUHSC_01422, and SAOUHSC_014213)) and the newly identified expressed segments colored according to the classification based on the transcriptional context (intragenic transcript (dark blue, *s595*), independent segment transcribed from its own promoter and with defined termination site (black, *s596*), 3′UTR following partial termination (yellow, *s597*)). The lower track shows for both strands the tiling array expression profiles (log2 scale) under selected iron-rich (TSB exp, TSB stat) and iron-limited (RPMI exp, RPMI stat, plasma exp) conditions. **(B)** Expression levels of *s596* and Fur-regulated genes *sirA* (Heinrichs et al., 1999) and *sfnA* (Cotton et al., 2009) in exponential growth phase under iron-rich (TSB, pMEM) and iron-limited (RPMI, plasma) conditions (Mäder et al., 2016).

In a proteome study of iron-responsive regulation in *S. aureus*, Friedman et al. (2006) observed that Fur-mediated regulation involves changes in the levels of central metabolism proteins, including lower amounts of TCA cycle enzymes under conditions of iron starvation, suggesting the presence of a functional RyhB analog in *S. aureus*. A very likely candidate, named S596, was identified in a large-scale transcriptome study (Mäder et al., 2016) (Figure 1A) based on its iron-dependent expression and *in silico* target analysis. S596 was co-regulated with known Fur targets (Figure 1B) and predicted to contain a Fur box in its promoter region. In line with these findings, the same sRNA was identified as the most highly upregulated sRNA (named Tsr25) in *S. aureus* USA300 grown in human serum compared to rich medium (TSB) (Carroll et al., 2016). Using CopraRNA2 (Wright et al., 2013), S596 was predicted to regulate, among others, the expression of genes encoding Fe-S cluster or heme containing proteins and TCA cycle enzymes (Mäder et al., 2016). Notably, S596 is conserved in staphylococci (Liu et al., 2018). Furthermore, Coronel-Tellez et al. (2022) described S596 as the only sRNA required for iron-limited growth of *S. aureus* in a competitive fitness assay with 48 sRNA mutants (Le Lam et al., 2017; Liu et al., 2018). Their study confirmed its regulation by Fur and demonstrated that S596 (renamed IsrR for iron-sparing response regulator) inhibits the translation of mRNAs encoding Fe-S cluster containing enzymes involved in anaerobic nitrate respiration. Moreover, IsrR was shown to be required for full virulence of *S. aureus* as a strain lacking IsrR is significantly attenuated in a mouse septicemia model (Coronel-Tellez et al., 2022).

In the present study, we combined proteomics-based identification of potential IsrR targets using *S. aureus* strains either lacking or constitutively expressing IsrR with an *in-silico* target prediction approach. In this way, we identified 21 IsrR targets including several Fe-S cluster and heme containing proteins, such as TCA cycle enzymes and *S. aureus* catalase encoded by *katA*. For most target mRNAs, the predicted interaction region of IsrR overlaps the ribosome binding site (RBS), thus implying that reduced protein amounts in the presence of IsrR result from translational repression. However, for some targets including *katA* base-pairing with IsrR outside the RBS is predicted. Determination of transcript half-lives indicates that IsrR can directly affect mRNA stability.

## Materials and methods

### Construction of bacterial strains

The bacterial strains and plasmids used in this study are listed in Table 1 and oligonucleotides are listed in Supplementary Table B. All experiments were performed with *S. aureus* HG001, a derivative of strain NCTC 8325 (Herbert et al., 2010), and isogenic mutants.

**Table 1:** Bacterial strains and plasmids used in this study.

*S. aureus* strains	Relevant genotype/characteristics	References
RN4220	Restriction-defective derivative of RN450 (*hsdR-, sauUSI-, agrA-, essC-, mntH*-), transformable	Kreiswirth et al., 1983
HG001	*rsbU*^+^-repaired and *tcaR*^−^-defective derivate of NCTC8325	Herbert et al., 2010
SGB007	HG001 Δ*isrR/s596::ermB*, *P_isrR_* Shine-Dalgarno-sequence *pgi*	This study
SGB009	HG001 Δ*fur::ermC*, *P_fur_* Shine-Dalgarno-sequence *fur*	This study
SGB010	HG001 Δ*fur::ermC*, *P_fur_* Shine-Dalgarno-sequence *fur* and Δ*isrR/s596::ermB*, *P_isrR_* Shine-Dalgarno-sequence *pgi*	This study
SGB011	HG001 pJLisrR	This study
SGB012	HG001 pJLctrl	This study
Plasmids		
pJL-sar-isrR = pJLisrR	pJL-sar plasmid with *isrR/s596* of HG001 under control of constitutive promoter *P*_*sarAP1*_ of *S. aureus* RN6734	This study
pJL-sar-ctrl = pJLctrl	pJL-sar plasmid without gene of interest under control of constitutive promoter *P*_*sarAP1*_ of *S. aureus* RN6734	This study

**Figure 2:**
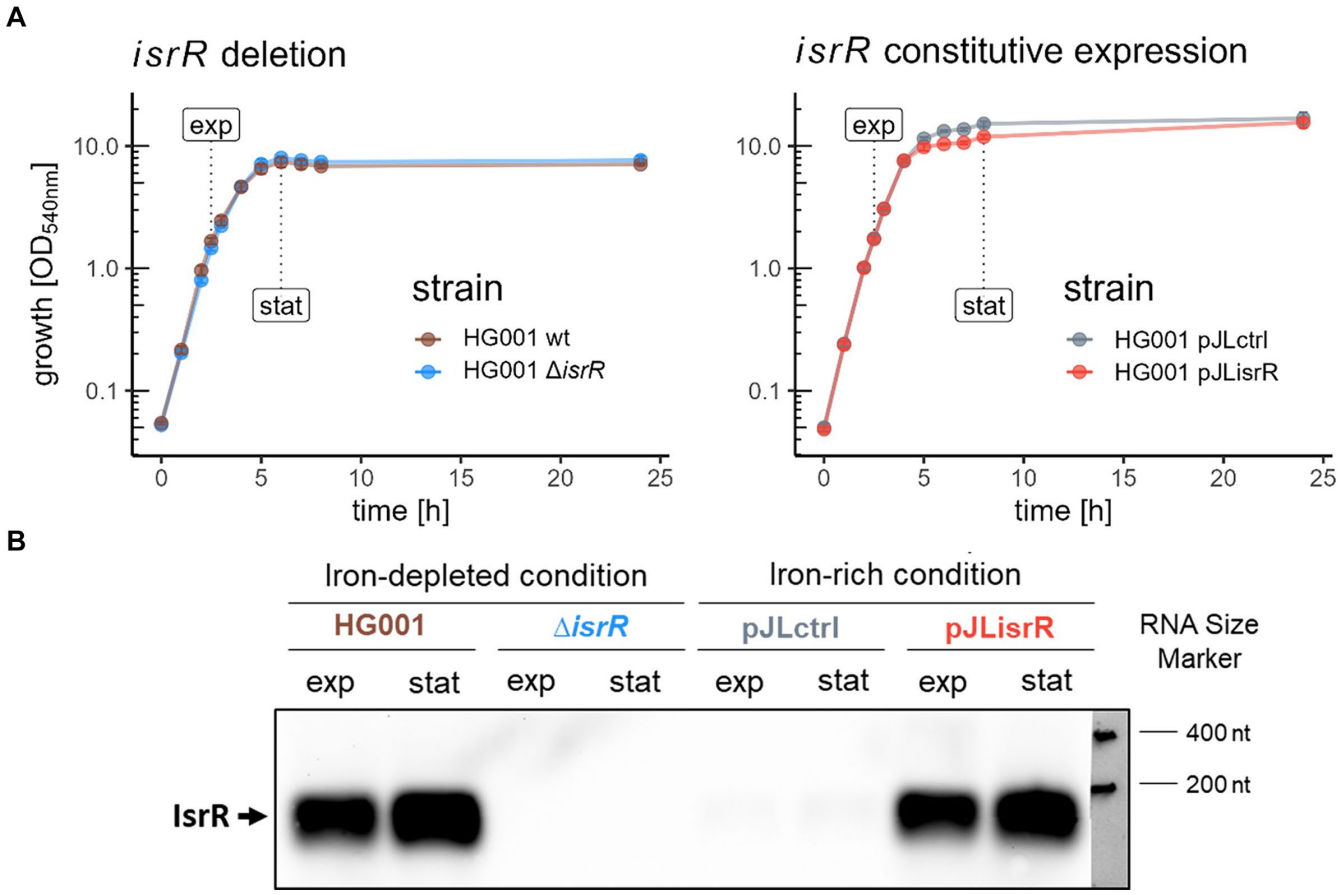
Growth and harvesting time points of samples for global proteome analysis and Northern blot analysis of IsrR levels. **(A)** In the first approach (left panel), *S. aureus* HG001 and its isogenic ∆*isrR* mutant were grown under IsrR-inducing iron-limited conditions (TSB + 600 μM DP); in the second approach (right panel), a constitutively *isrR*-expressing strain (HG001 pJLisrR) is compared to the empty vector control (HG001 pJLctrl) under iron-rich conditions (TSB). **(B)** Northern blot analysis of IsrR sRNA. RNA samples were obtained at the same time points and under the same cultivation conditions as the proteome samples (upper panel). For each sample, 4 μg of total RNA was loaded per lane.

The mutagenesis plasmids were derived from pBASE6 (Geiger et al., 2012) and constructed in *E. coli* DC10B via the Sequence and Ligase Independent Cloning (SLIC) method (Li and Elledge, 2012) using the indicated primers (Supplementary Table B) for PCR amplification. Details are described in the Supplementary Data. Mutagenesis was performed according to the procedure described by Bae and Schneewind (2006), and the complete *isrR* and/or *fur* coding sequences were replaced with the corresponding resistance cassettes. The resulting mutant strains SGB007 (HG001 Δ*isrR*), SGB009 (HG001 Δ*fur*) and SGB010 (HG001 Δ*fur* Δ*isrR*) were verified by PCR, Northern blot analysis, and DNA sequencing. The sRNA expression plasmid and the respective control plasmid were constructed in *E. coli* Stellar^™^ using the vector pJL-sar-GFP (Liese et al., 2013). The *gfpmut2* sequence in pJL-sar-GFP was replaced with the *isrR* coding sequence to generate pJLisrR using the indicated primers. For pJLctrl, the *gfpmut2* sequence was deleted from pJL-sar-GFP. After verification by DNA sequencing, the resulting plasmids were transferred to the restriction-defective *S. aureus* strain RN4220 and then introduced into *S. aureus* HG001 by electroporation. The resulting strain SGB011 with constitutive IsrR expression and the corresponding control strain SGB012 were verified by Northern blot analysis (Figure 2B).

### Media and growth conditions

Bacteria were cultivated in tryptic soy broth (TSB; BD, Franklin Lakes, NJ, United States) medium and in iron-depleted TSB medium (TSB-DP). Iron depletion was achieved by addition of the iron chelator 2,2′-dipyridyl (DP) at a concentration of 600 μM, followed by incubation for at least 1 h. The *S. aureus* main cultures were inoculated with an exponentially growing overnight culture to an optical density at 540 nm (OD_540_) of 0.05 and grown aerobically at 37°C. Antibiotics were used at the following concentrations: ampicillin 100 μg/mL for *E. coli*; chloramphenicol 10 μg/mL and tetracycline 5 μg/mL for *S. aureus*.

For the spot test assay, *S. aureus* HG001 wild type and HG001 Δ*isrR* were cultured in triplicates in TSB-DP, and 5 μL of exponentially growing cells (OD_540_ of 1.0) were spotted in dilution series from 10^−1^ to 10^−6^ onto a TSB-DP agar plate. The plate was incubated overnight at 37°C.

### Sample harvest and preparation for analysis by mass spectrometry

Bacterial strains were cultivated as described above and cells were harvested in exponential and stationary growth phase by centrifugation (4°C, 10,000 × g, 3 min) and cell pellets were frozen in liquid nitrogen. For mechanical disruption of bacterial cells, frozen cell pellets were suspended in 100 μL 20 mM 2-[4-(2-hydroxyethyl)piperazin-1-yl]ethanesulfonic acid (HEPES) + 5% (w/v) sodium dodecyl sulfate (SDS), treated in a bead mill (Retsch GmbH, Haan, Germany; 3 min, 30 Hz) and the cell powder was resuspended in 400 μL 20 mM HEPES. The cell lysates were treated with Pierce^™^ Universal Nuclease (Pierce, Thermo Fisher Scientific, MA, United States; 2.5 U, 4 mM MgCl_2_, 20 min, 37°C) followed by ultra-sonication for 5 min in an ultrasonic bath (Sonorex, Bandelin, Germany). To remove cell debris, samples were centrifuged (30 min, 17,000 × g, RT). Protein concentrations were determined using a Micro BCA^™^ Protein Assay Kit (Pierce, Thermo Fisher Scientific, MA, United States).

Tryptic digestion of proteins and peptide purification were performed as described in Blankenburg et al. (2019) with minor adjustments: 4 μg protein mixture per sample were incubated with 80 μg of hydrophilic (GE Healthcare, Little Chalfont, United Kingdom) and hydrophobic (Thermo Fisher Scientific, MA, United States) carboxylate-modified magnetic SeraMag Speed Beads in 70% (v/v) acetonitrile (ACN) and shaken in a ThermoMixer (1,400 rpm, RT, 18 min). Beads were washed twice with 70% (v/v) ethanol and once with 100% (v/v) ACN. For tryptic digestion, beads were incubated with 160 ng trypsin (Promega Corporation, Wisconsin, United States) in 20 mM ammonium-bicarbonate buffer (37°C, 16 h). By adding ACN to a final concentration of 95% (v/v) and shaking in a ThermoMixer (18 min, 1,400 rpm, RT), the digestion was stopped. Beads were washed with 100% (v/v) ACN and peptides were eluted from the beads with 2% (w/v) dimethyl sulfoxide and ultra-sonication for 5 min. Finally, the samples were resuspended in buffer A (4% (v/v) ACN, 0.2% (v/v) acetic acid) and stored at −20°C.

### Mass spectrometric measurements and data analysis

Tryptic peptide solutions were separated for LC–MS/MS analysis on an Ultimate 3,000 nano-LC system (Thermo Fisher Scientific, MA, United States) and analysed in data-independent acquisition (DIA) mode on a Q Exactive^™^ HF mass spectrometer (Thermo Fisher Scientific, MA, United States). For further details, see Supplementary Tables C, D.

DIA mass spectrometry (MS) data was searched against a *S. aureus* NCTC8325 protein data base (fasta file downloaded December 2017 from *Aureo*Wiki (Fuchs et al., 2018) comprising 2,853 staphylococcal protein sequences). The RsbU sequence was replaced by the *S. aureus* Newman RsbU sequence and three contaminant control protein sequences (lysostaphin, benzonase, trypsin) and the sequence of the allele replacement marker ErmB were added. The search was performed using the Spectronaut software (v16.0.220606.53000; Biognosys AG, Schlieren, Switzerland) with settings described in Supplementary Table E in the directDIA analysis mode in accordance to Michalik et al. (2017). MS data from the iron-limitation experiment and the constitutive expression experiment were analyzed separately. The mass spectrometry proteomics data have been deposited to the ProteomeXchange Consortium via the PRIDE (Perez-Riverol et al., 2022) partner repository with the dataset identifier PXD045218.

Global median-normalized Spectronaut-processed data was further analyzed in R (v4.1.2). All R packages used for analysis and visualization are listed in Supplementary Table F. For global proteome analysis, only protein groups identified with at least two peptides were considered. 1,486 and 1,612 protein groups were identified for the experiments under iron-limited and iron-sufficient conditions, respectively. 1,416 protein groups were commonly identified in both experimental setups. For visualization purposes, protein groups were split into the respective proteins. Peptide-based ROPECA (Suomi and Elo, 2017) statistics were calculated protein-wise for each mutant/constitutive expressing strain compared to HG001 wild type/control strain. Proteins were assumed to differ significantly in abundance between conditions if the FDR-adjusted *p*-value (*q*-value) was less than 0.05 and the absolute fold change was at least 1.5. Protein levels were estimated using the maxLFQ algorithm (Cox et al., 2014).

### *In silico* prediction of IsrR targets

IsrR targets of *S. aureus* HG001 were predicted exploiting the CopraRNA2 (v2.1.4 using IntaRNA 2.4.1; Wright et al., 2013) RNA web tool (v5.0.7.; Raden et al., 2018). For higher sensitivity, different sets of search parameters were used: sequence window size (300 bp upstream to 300 bp downstream of the start/stop codon, 200 bp upstream to 100 bp downstream of the start/stop codon), interaction region (start or stop codon), and the organism set. The organism sets were selected based on blastn (Camacho et al., 2009) hits for IsrR orthologues in the data base of representative RefSeq genomes (30.06.2022) of Staphylococcaceae (taxid:90964). Only organisms with completely assembled genomes were considered. In a first set IsrR orthologues with an identity of at least 85% and in a second set IsrR orthologues with at least 90% coverage were included. To improve the prediction of *S. aureus* targets in the organism sets, four additional *S. aureus* strains from *Aureo*Wiki (Fuchs et al., 2018) were supplementary added to the sets. The respective organisms and orthologues are listed in Supplementary Table G.

Further processing of the prediction was performed in R (v4.1.2). All R packages used for analysis and visualization are listed in Supplementary Table F. Targets with an FDR-adjusted *p*-value (*q*-value) ≤ 0.05 were selected. Since most sRNA interactions occur at the RBS located in the 5′-UTR of the target mRNA (Desnoyers et al., 2013), we semi-automatically curated the list of targets with predicted IsrR 3′-target-interactions. For that, the predicted position of the interaction site was compared to the likely Shine-Dalgarno (SD) sequence (−15 to −4 bp relative to the start codon) of the next same-stranded downstream gene. We then considered those downstream genes as predicted 5′-targets. For further analyses, we omitted remaining 3′-targets.

For classification of putative regulatory mechanisms by IsrR, putative interaction of IsrR with the target RBS (−20 to +15 bp relative to start codon) was determined based on the localization of the predicted IsrR interaction site.

### RNA preparation and northern blot analysis

Samples for RNA preparation were harvested at the same time points as described before. About 15 OD_540_ units were harvested after addition of 1/3 volume of frozen killing buffer (20 mM Tris/HCl [pH 7.5], 5 mM MgCl_2_, 20 mM NaN_3_) by subsequent centrifugation for 3 min at 10,000 × g and 4°C. After discarding the supernatant, cell pellets were frozen in liquid nitrogen and stored at −80°C.

Total RNA was prepared by acid-phenol extraction after mechanical cell disruption as described previously (Nicolas et al. 2012). For Northern blot analysis, 4 μg of total RNA per sample was separated under denaturing conditions in 1.2% (w/v) agarose gels prepared by mixing 1.2 g agarose, 74 mL water, 10 mL 10× MOPS, 10 mL formaldehyde (37% (v/v)) and 5 μL ethidium bromide. After electrophoretic separation with 1× MOPS as running buffer, RNA quality and equal loading were controlled by visualization of the 16S and 23S rRNA under UV light. Subsequently, the RNA was transferred by vacuum blotting onto a positively charged nylon membrane (Pall Corporation, Pensacola, FL, United States) and cross-linked (120 mJ/cm^2^, Stratalinker UV Crosslinker 1800, Agilent Technologies, Santa Clara, CA, United States). The membranes were pre-hybridized for 1 h at 68°C and hybridized overnight at 68°C with continuous rotation in 15 mL of hybridization solution containing 50% (v/v) formamide, 5× SSC, 0.02% (w/v) SDS, 0.1% (w/v) N-lauroyl sarcosinate, 2% (w/v) blocking reagent and 1 μg of digoxygenin (DIG)-labelled RNA probe. DIG-labelled RNA probes were synthesized by *in vitro* transcription with T7 RNA polymerase and gene-specific PCR products as template. Primer sequences are listed in Supplementary Table A. After washing, the membranes were incubated for 30 min at room temperature with 1:10,000 diluted anti-DIG-AP F_ab_ antibody fragments (Roche, Grenzach-Wyhlen, Germany) and washed again. Then, the pH was adjusted to 9.5, the optimal pH for the antibody-conjugated alkaline phosphatase, and the chemiluminescent substrate CDP-*Star*^™^ (Thermo Fisher Scientific, MA, United States) was added. Chemiluminescence signals were detected using a ChemoCam Imager (Intas Science Image Instruments, Göttingen, Germany).

### Determination of mRNA half lives

To inhibit transcription initiation, rifampicin (100 μg/mL) was added to *S. aureus* cultures growing aerobically in a water bath, at the same time points in exponential and stationary phase as for the proteome analysis. Samples were harvested either immediately (*t*_0_) or 1, 2, 4, 6, and 8 min after adding rifampicin. Sample harvesting, preparation of total RNA and Northern Blot analysis were carried out as described above. The quantification of the specific chemiluminescence signals was performed using the Studio Lite Image Studio^™^ software version 5.2 (LI-COR Biosciences, Lincoln, United States). Values were normalized to the initial value (*t*_0_ = 100%) of the time-series. The regression model $y\;=a*e^{-bx}$ was used to determine the mRNA half-life (*t*_1/2_).

### Aconitase assay

To prepare the protein samples of *S. aureus* strains for the aconitase assay, 15 OD_540_ units were harvested by centrifugation (3 min, 4°C, 10,000 × g). After washing with 1 mL 50 mM Tris–HCl (pH 8.0), the cells were pelleted again, frozen in liquid nitrogen and stored at −80°C. Cells were resuspended in 400 μL 50 mM Tris–HCl and treated with 10 μg lysostaphin (AMBI PRODUCTS LLC, NY, United States) and 2.5 units benzonase (Pierce, Thermo Fisher Scientific, MA, United States; 4 mM MgCl_2_) followed by incubation at 37°C for 30 min. To remove cell debris, samples were centrifuged (5 min, 13,000 × g, 4°C). Subsequently, 400 μL of the supernatant were carefully transferred to a fresh reaction tube placed on ice.

Aconitase enzyme activity was analyzed by a UV-spectrophotometric assay measuring the conversion of isocitrate to *cis*-aconitate as introduced by Dingman and Sonenshein (1987). For this, 100 μL of freshly prepared protein extract was incubated with 100 μL of activation buffer containing 8 mM DTT and freshly prepared 0.8 mM Mohr’s salt [(NH_4_)_2_Fe(SO_4_)_2_(H_2_O)_6_] in 50 mM Tris–HCl (pH 8.0) for 10 min at 25°C as a source of iron for reconstitution of the iron–sulfur cluster in the aconitase enzyme as introduced by Dingman and Sonenshein (1987). Subsequently, aconitase activity was determined as change in absorbance [∆*A*_240nm_/min] and normalized to the protein concentration. The assay was carried out in duplicate for two biological replicates.

### Disk diffusion assay of hydrogen peroxide sensitivity

To test the sensitivity of *S. aureus* strains against exogenous oxidative stress elicited by hydrogen peroxide (H_2_O_2_), a disk diffusion assay was performed as introduced by Frees et al. (2003). *S. aureus* HG001 wild type, HG001 Δ*isrR*, HG001 pJLisrR and HG001 pJLctrl were cultured in TSB until reaching an OD_540_ of 1. Subsequently, 100 μL of each exponential culture was plated onto both TSB and TSB-DP agar plates. In the case of TSB-DP plates, 2 μL of a 30 mM DP stock solution were added to the cells to deplete available iron. For plasmid-containing strains, chloramphenicol (10 μg/mL) was included in the media. When the plates were dry, a sterilized paper disc with a diameter of 6 mm was positioned at the center of each plate and gently pressed onto the agar surface. Then, 10 μL of a 3% (v/v) H_2_O_2_ solution was carefully pipetted onto the center of the paper disc. The plates were incubated at 37°C for 24 h. The antimicrobial activity of H_2_O_2_ was evaluated by measuring the diameter of the zones where growth was inhibited (zone of inhibition) surrounding the paper discs. The assay was carried out in duplicate for four biological replicates.

## Results

### Experimental identification of potential IsrR targets

In order to identify potential IsrR targets, two different experimental approaches were used. In the first setup, proteome profiles of the *S. aureus* HG001 strain were compared with those of the isogenic Δ*isrR* mutant SGB007. The strains were grown in TSB medium under iron-limitation elicited by the addition of the divalent metal ion chelator 2,2’-Dipyridyl (DP) (600 μM), because transcription of *isrR* is repressed by Fur in the presence of iron. DP is widely used to induce iron limitation in *S. aureus* (Allard et al., 2006; Friedman et al., 2006; Torres et al., 2010) and in agreement with this, addition of iron almost completely abolished the effect of DP on the growth of *S. aureus* in TSB (Supplementary Figure S1C). Under iron-limited conditions, IsrR is strongly up-regulated as demonstrated by Northern blot analysis (Supplementary Figures S1A,B). Surprisingly, growth of the Δ*isrR* mutant was not impaired in TSB-DP liquid cultures (Figure 2A). However, in dilution series on TSB-DB agar plates the mutant colonies were noticeably smaller and displayed slightly lower numbers than those of the parental strain (Supplementary Figure S1D), supporting spot test assays on BHI medium by Coronel-Tellez et al. (2022).

In the second approach, the effects of IsrR on the proteome were analyzed under iron-replete conditions using a strain in which *isrR* is constitutively expressed from a plasmid under the control of the *sarA* P1 promoter (Cheung et al., 1998; Liese et al., 2013). When grown in TSB medium, this strain (SGB011) exhibits comparable IsrR levels as the HG001 wild-type under iron-limited conditions (TSB-DP), both during exponential growth and in stationary phase (Figure 2B). In line with the growth behavior under iron-limitation, absence or presence of IsrR only modestly affected growth, namely in the early stationary phase (Figure 2A).

**Figure 3:**
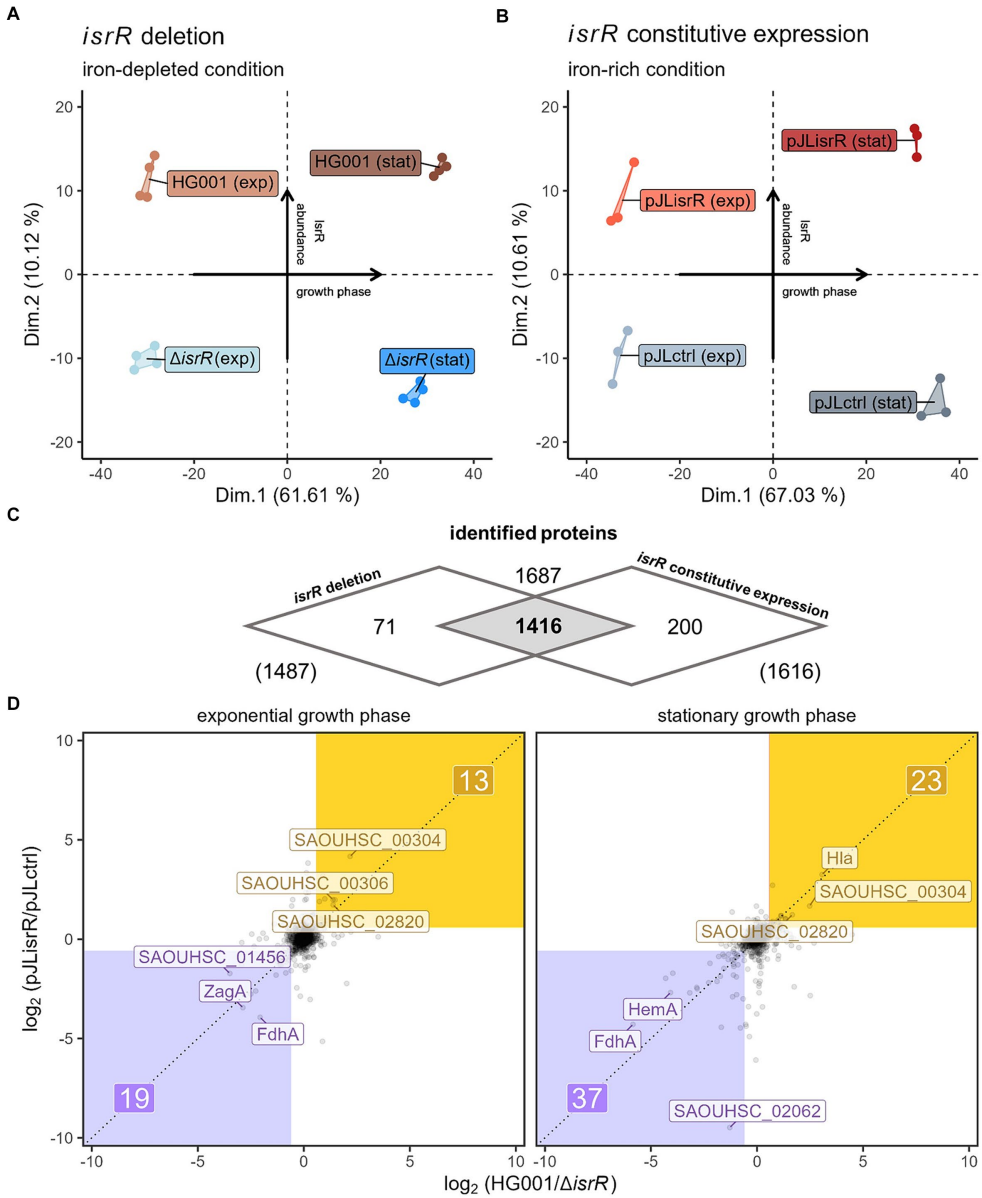
Comparison of global differences in proteome profiles driven by IsrR. **(A)** Principal component analyses of proteomic profiles of HG001 wild type and ∆*isrR* mutant in exponential and stationary growth phase under iron-depleted conditions and **(B)** constitutively *isrR*-expressing HG001 and HG001 carrying the control vector in exponential and stationary growth phase under iron-rich conditions. **(C)** Venn diagram summarizing the numbers of identified proteins for the two proteome analyses. **(D)** Correlation plot of IsrR-driven differences in protein abundances (log_2_ scale) between both experimental approaches (*x*-axis: approach 1; *y*-axis: approach 2) in exponential and stationary growth phase. Negatively affected proteins (FC ≤ 1.5 in both experiments and geometric mean of *q*-values ≤0.05) are depicted in purple, positively affected proteins are depicted in yellow. For each growth phase and group, the *Top3* of affected proteins are labeled.

The combination of both experimental approaches was chosen to analyze IsrR-dependent changes in the abundance of individual proteins under different growth conditions in terms of iron availability and Fur regulon expression. With this combination we intended to increase the reliability of experimental identification of potential IsrR targets. For both analyses, samples were taken in the exponential and stationary growth phases (Figure 2A). Cell pellets were processed and samples were analyzed by mass spectrometry as described in the Materials and Methods section. Out of 2,852 annotated protein coding sequences, 1,487 proteins (∼52%) were identified in the analysis of *S. aureus* HG001 wild-type versus Δ*isrR* mutant under iron-depleted conditions and 1,616 proteins (∼57%) in the analysis of HG001 pJLisrR versus HG001 pJLctrl under iron-rich conditions, respectively (Supplementary Table S1; Figure 3C).

To visualize the influence of IsrR on overall protein profiles, a principal component analysis of the normalized maxLFQ protein intensities was performed (Figures 3A,B). In both data sets, the first principal component separated the samples according to the growth phase, reflecting the pronounced differences in gene expression between exponential and stationary phase cells (Mäder et al., 2016). It explained ∼62% and ∼67%, respectively, of the total variances. Notably, the second axis describing 10.1% and 10.6% of the total variances led to separation according to the *isrR* expression status, indicating a profound and similar impact of IsrR on the proteome profile under both experimental conditions.

Correlation plots comparing log_2_ protein ratios of the 1,416 proteins commonly identified in both experimental set-ups (Figure 3C) between *isrR*-expressing and non-expressing strains revealed that the presence of IsrR caused a considerable number of consistent changes in protein levels (Figure 3D). In the exponential growth phase, 19 proteins were more than 1.5-fold less abundant in both approaches with a combined *p*-value ≤0.05 (geometric mean of FDR-adjusted *p*-values) and in the stationary phase, 37 proteins were identified as less abundant in both setups in the presence of IsrR compared to its absence. Proteins with lower abundance in the presence of IsrR potentially correspond to direct IsrR targets regulated through the classical mode of sRNA action, i.e., inhibition of translation by obstructing the RBS (Desnoyers et al., 2013). Notably, FdhA was less abundant in both approaches in exponential growth and stationary phase, which is in agreement with the identification of FdhA as a direct IsrR target by Coronel-Tellez et al. (2022).

Surprisingly, 13 proteins (exponential growth) and 23 proteins (stationary phase) were more abundant in the presence of IsrR in both approaches, which could result from direct enhancement of translation by IsrR. However, sRNA-driven effects on particular proteins can also be indirect, caused by, e.g., altered activities of regulatory proteins (Georg et al., 2020).

### Integration of experimental data and IsrR target prediction

To distinguish the direct targetome of IsrR from changes of the proteome indirectly driven by IsrR, experimentally determined target candidates were integrated with computationally predicted targets based on sRNA-mRNA interactions.

**Figure 4:**
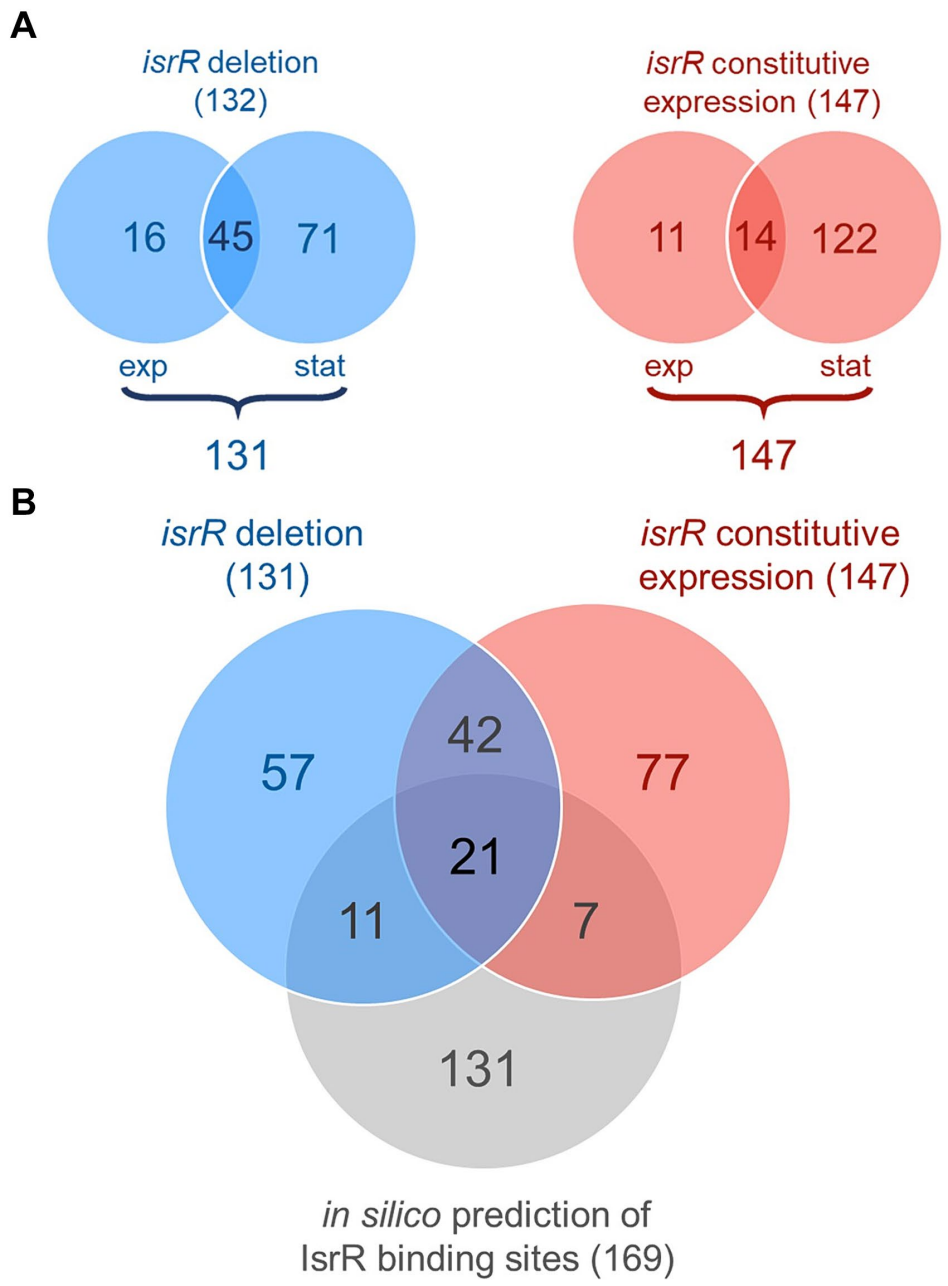
Overview of IsrR target candidates. **(A)** Venn diagrams of differentially abundant proteins. Numbers of significantly different abundant proteins (ROPECA: absolute fold change of ≥1.5 and *q*-value ≤0.05) between the HG001 wild type and ∆*isrR* mutant under iron-limited conditions are depicted in blue; numbers of significantly different abundant proteins between the constitutively *isrR*-expressing strain and the empty vector control are depicted in red. Proteins, which are in at least one growth phase significantly different abundant but not regulated in opposite directions between the exponential and stationary growth phase, are considered as candidates in the particular experiment (numbers below the diagrams). Of the 132 proteins displayed in the left diagram, one was discordant between the growth phases and therefore excluded from further analysis. **(B)** Venn diagram of experimental and *in silico* predicted IsrR target candidates. Experimental candidates: number of proteins with significantly different abundance in at least one growth phase but concordance between both growth phases (ROPECA: absolute fold change of ≥1.5 and *q*-value ≤0.05) in the respective experiment. The first experimental setup comparing the HG001 wild-type and ∆*isrR* mutant strains under iron-limited conditions is depicted in blue, the second experimental setup comparing the constitutively *isrR*-expressing strain with the empty vector control is depicted in red. CopraRNA2-based *in silico* prediction is depicted in gray. Proteins with concordant responses to IsrR presence are considered in the overlap between both experimental set-ups. The one discordant protein (PflB) with predicted IsrR binding site is counted separately for the two experiments.

The set of experimental target candidates was generated based on the concordant results of the two experimental approaches. For that, we first determined the set of potential IsrR targets for each experimental approach. Proteins showing different abundances between the *isrR*-expressing condition and non-expressing condition in the exponential and/or stationary phase were categorized as candidates in the particular experiment. The two sampling time points were not distinguished because the bacterial cultures are assumed to exhibit physiological differences between iron-limited and iron-rich conditions, which might be associated with differences in growth phase-specific protein patterns. Between HG001 and the Δ*isrR* mutant in TSB-DP, 132 of the 1,416 commonly identified proteins (Supplementary Table S1; Figure 4A) showed significantly different abundance in at least one growth phase (ROPECA: absolute fold change of ≥1.5 & *q*-value ≤0.05), of which one protein was discordant between the two growth phases and therefore excluded from further analysis. The abundance of 147 of the 1,416 commonly identified proteins (Supplementary Table S1; Figure 4A) was significantly different between the strain constitutively expressing *isrR* under iron-replete conditions (HG001 pJLisrR) and the control strain with the empty vector (HG001 pJLctrl). 63 proteins were consistently identified in both experimental approaches as displaying IsrR-dependent differences in abundance and thus considered as experimental candidates for IsrR targets (Figure 4B; Supplementary Table S1).

As a complementary method to identify potential IsrR targets, a CopraRNA2-based relaxed *in silico* target prediction was performed. Since bacterial sRNAs bind to mRNA targets through short regions of imperfect complementarity, *in silico* target prediction is challenging, even though it can be improved by comparative target prediction for conserved sRNAs (Backofen and Hess, 2010; Wright et al., 2013) as exploited by CopraRNA2. Because of the challenges in the prediction of sRNA targets, the *in silico* analysis was performed with rather relaxed criteria to increase the sensitivity of the prediction. We combined analyses performed with two different search window sizes (−300 bp to +300 bp and −200 bp to +100 bp relative to the start/stop codon of all protein coding genes) and four different organism sets (Material and Methods; Supplementary Table G). Additionally, we performed searches for sRNA-mRNA interactions at the 5′-region and the 3′-region of transcripts, i.e., around the start and the stop codon. Thereby, initially 236 5′-targets and 342 3′-targets were predicted in at least one prediction set (*q*-value ≤0.05). However, for 51 of the predicted 3′-targets the calculated interaction sites mapped at a sequence likely covering the SD sequence (−15 to −4 bp relative to the start codon) of the gene located downstream of the initially predicted target. Of these 51 putative 5′-targets, 24 were also predicted as 5′-targets in the initial target prediction, whereas 27 have most probably escaped detection in the initial target prediction; they were considered to be falsely non-annotated putative 5′-targets and therefore included in the set of predicted 5′-targets. Since most sRNA-target interactions occur at the RBS (Desnoyers et al., 2013), the 263 curated 5′-targets (Supplementary Table S2) were considered for the subsequent integration of *in silico* and experimentally predicted targets, and the remaining 3′-targets were discarded. For 169 of the 263 potential IsrR targets, the corresponding proteins were covered in both proteomics experiments (Figure 4B).

By combining the proteome data with the *in silico* target prediction, 21 very likely IsrR targets were identified (Figure 4B), which were targets predicted *in silico* and displaying IsrR dependent changes in abundance in both cultivation setups. The vast majority of these 21 targets (19/21) were negatively affected by IsrR based on the protein patterns observed (Table 2). Of these, nine proteins are related to known targets of Fur-regulated sRNAs, which are Fe-S cluster or heme containing proteins (CitB, FdhA, MiaB, SAOUHSC_02760, KatA), proteins involved in heme biosynthesis (HemA, HemY) and TCA cycle (CitB, SdhA, SucA). For two Fe-S cluster proteins identified by our analysis, formate dehydrogenase FdhA and glutamate synthase SAOUHSC_02760 (Clusters of Orthologous Genes (COG) symbol: GltB2), translational repression by IsrR was previously demonstrated by Coronel-Tellez et al. (2022). Two proteins were identified as potentially positively regulated by IsrR, SAOUHSC_00827 and SAOUHSC_00304 (Table 2). The predicted and experimentally demonstrated (specifically for *fdhA* and SAOUHSC_02760) IsrR binding sites on the 21 target mRNAs are summarized in Supplementary Table H.

**Table 2:** The very likely IsrR targets, negatively regulated (19 out of 21) or positively regulated (2 out of 21) by the sRNA, including fold changes and *q*-values for both experimental approaches.

Protein	Fold change ∆*isrR*/HG001 (*q*-value)	Fold change pJLisrR/pJLctrl (*q*-value)	IsrR-binding predicted in RBS region
Negatively regulated by IsrR			
CitB (SAOUHSC_01347)	exp: 2.5 (3.9e−39) stat: 4.9 (4.6e−50)	exp: −3.5 (4.2e−16) stat: −4.6 (6.9e−50)	x
SucA (SAOUHSC_01418)	exp: 2.1 (6.1e−12) stat: 3.5 (4.0e−51)	exp: −2.1 (6.6e−08) stat: −1.8 (5.6e−28)	
KatA (SAOUHSC_01327)	exp: 1.4 (1.4e−09)† stat: 2.9 (2.9e−31)	exp: −1.7 (1.9e−03) stat: −2.8 (8.3e−26)	
SdhA (SAOUHSC_01104)	exp: 1.6 (8.1e−02)† stat: 4.6 (3.3e−29)	exp: −2.1 (3.8e−05) stat: −2.3 (4.1e−24)	X
Rnd2 (SAOUHSC_02525)	exp: 1.4 (3.4e−10)† stat: 1.9 (2.4e−28)	exp: −1.7 (3.7e−03) stat: −1.7 (3.7e−17)	
AcsA (SAOUHSC_01846)	exp: 1.3 (1.0e+00)† stat: 2.2 (1.1e−25)	exp: −1.7 (8.3e−02)† stat: −2.4 (1.2e−29)	X
FdhA (SAOUHSC_02582)	exp: 4.2 (8.2e−03) stat: 57.7 (1.0e−05)	exp: −15.3 (2.8e−16) stat: −19.6 (1.7e−31)	X
HemY (SAOUHSC_01960)	exp: 4.8 (1.9e−12) stat: 7.1 (7.8e−13)	exp: −6.1 (4.8e−05) stat: −5.6 (4.1e−12)	X
SAOUHSC_00875	exp: 1.3 (3.1e−04) stat: 2.6 (3.7e−20)	exp: −1.3 (5.8e−01)† stat: −2.0 (7.3e−15)	X
RocF (SAOUHSC_02409)	exp: −1.1 (1.0e+00)† stat: 3.7 (6.3e−19)	exp: −1.3 (8.0e−01)† stat: −4.4 (3.5e−19)	
MiaB (SAOUHSC_01269)	exp: 2.3 (2.7e−04) stat: 2.2 (1.4e−03)	exp: −2.1 (6.6e−04) stat: −2.0 (4.0e−14)	X
FadE (SAOUHSC_00198)	exp: 1.2 (1.0e+00)† stat: 1.5 (6.5e−04)	exp: −1.6 (2.1e−01)† stat: −2.1 (7.4e−15)	X
SAOUHSC_02003	exp: 2.2 (6.4e−04) stat: 15.7 (4.8e−07)	exp: −2.4 (6.9e−02)† stat: −3.3 (6.6e−08)	X
SAOUHSC_02760	exp: 1.5 (2.6e−01)† stat: 2.0 (2.3e−06)	exp: −1.7 (1.1e−01)† stat: −1.9 (2.0e−10)	X
HemA (SAOUHSC_01776)	exp: 7.6 (5.9e−06) stat: 16.9 (2.7e−07)	exp: −2.4 (3.8e−01)† stat: −6.4 (9.7e−05)	
CcpE (SAOUHSC_00679)	exp: 1.5 (5.9e−01)† stat: 2.1 (4.3e−09)	exp: −1.2 (1.0e+00)† stat: −2.1 (8.1e−07)	X
SAOUHSC_02861	exp: 1.4 (2.5e−02)† stat: 2.1 (3.7e−06)	exp: −1.7 (4.3e−01)† stat: −2.6 (2.5e−07)	X
BstA (SAOUHSC_03028)	exp: 2.1 (1.4e−03) stat: 3.3 (3.3e−05)	exp: −2.5 (5.2e−01)† stat: −3.6 (6.0e−04)	X
DtpT (SAOUHSC_00738)	exp: 2.1 (4.4e−04) stat: 1.8 (7.3e−04)	exp: −2.5 (3.8e−01)† stat: −1.8 (9.2e−04)	X
Positively regulated by IsrR			
SAOUHSC_00304	exp: −4.5 (8.2e−08) stat: −5.6 (5.2e−10)	exp: 18.0 (3.4e−03) stat: 3.2 (1.8e−06)	
SAOUHSC_00827	exp: −1.4 (8.8e−01)† stat: −2.1 (5.0e−03)	exp: 2.4 (1.0e+00)† stat: 1.6 (4.9e−02)	

^†^The dagger indicates a non-significant difference in protein level in the respective growth phase.

### IsrR-mediated downregulation of TCA cycle enzymes

In the course of the iron-sparing response, bacterial cells downregulate iron-requiring, non-essential pathways such as the TCA cycle (reviewed in, e.g., Oglesby-Sherrouse and Murphy, 2013). Two TCA cycle enzymes, aconitase and succinate dehydrogenase (SDH), are among the common targets of Fur-regulated sRNAs, including FsrA of *B. subtilis* (Gaballa et al., 2008). In our study, three TCA cycle enzymes were identified as very likely IsrR targets (Table 2), namely aconitase (CitB), SDH subunit SdhA, and 2-oxoglutarate dehydrogenase subunit SucA.

Aconitase is a 4Fe-4S cluster enzyme that converts citrate to isocitrate. By measuring aconitase enzymatic activity in the constitutively *isrR*-expressing strain and the empty vector strain under iron-rich conditions as well as in the HG001 wild type and the isogenic *isrR* mutant under iron limitation, IsrR-dependent decrease in CitB synthesis was matched by corresponding decreases in aconitase activity (Supplementary Figure S2). In addition to reduced protein synthesis under iron-limiting conditions, loss of the Fe-S cluster results in an inactive enzyme that acts as an mRNA-binding protein in different organisms (reviewed in Kiley and Beinert, 2003). This regulatory function of aconitase has recently been confirmed for *S. aureus* (Barrault et al., 2024).

**Figure 5:**
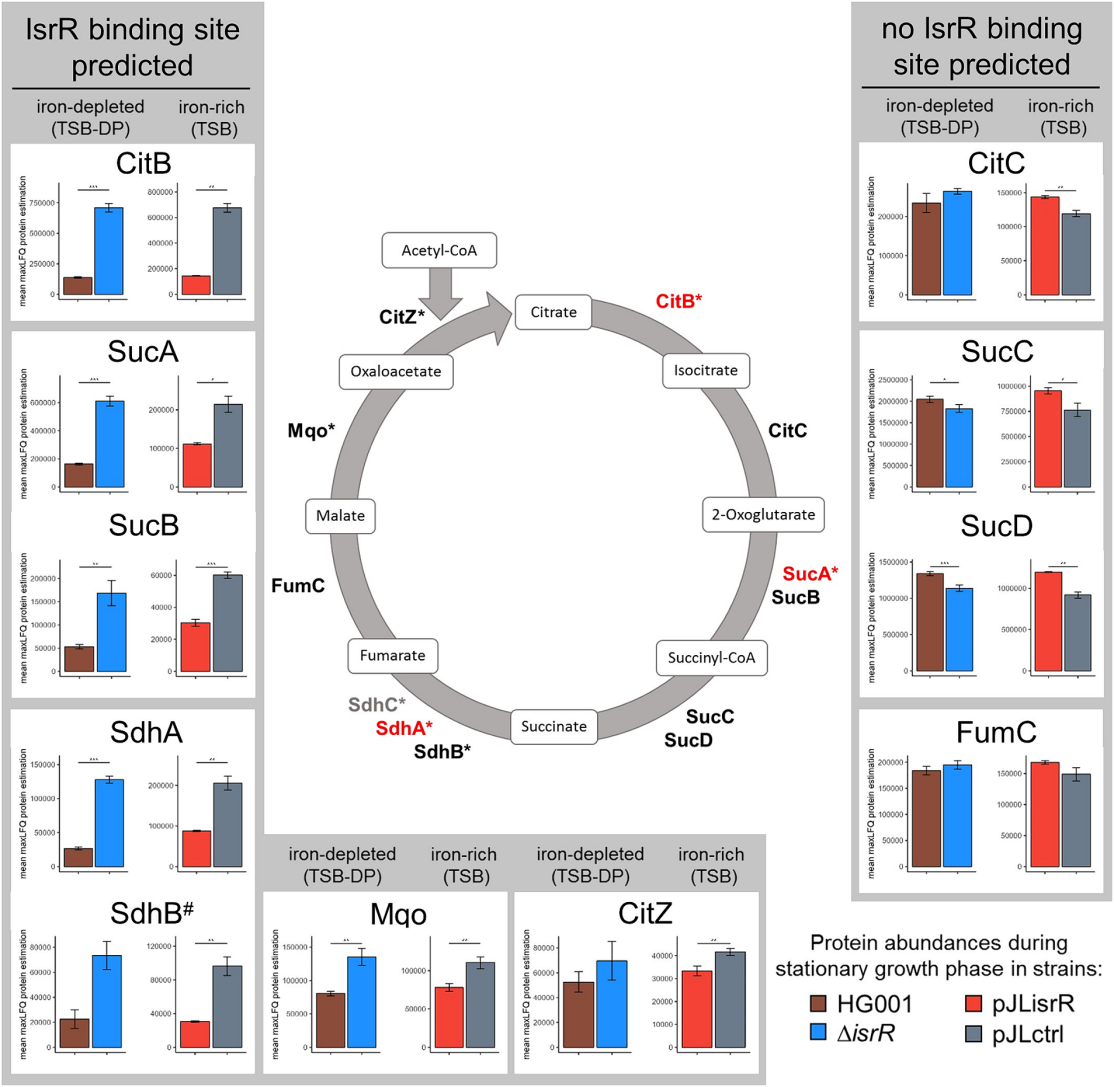
A simplified metabolic model of the TCA cycle with proteins identified as IsrR targets shown in red. The asterisk indicates whether an IsrR binding site was predicted for the transcript of the respective protein. Proteins not detected in the proteome analysis are highlighted in gray. The bar charts show the amount (mean maxLFQ protein level) of each TCA cycle protein in stationary phase between *isrR*-expressing (HG001, pJLisrR) and non-expressing strains (∆*isrR*, pJLctrl). Error bars represent the standard deviation of the biological replicates. Statistics: Welch-*t*-test on protein levels (*p* < 0.001***, *p* < 0.01**, and *p* < 0.05*).

The SDH complex encoded by the *sdhCAB* operon consists of three subunits, the flavoprotein SdhA, the Fe-S cluster containing SdhB and the heme-binding cytochrome b558 SdhC. In *B. subtilis*, FsrA binds to the leader region of the *sdhCAB* mRNA, most probably regulating the translation of *sdhC* and subsequently the stability of the tricistronic transcript (Gaballa et al., 2008). Similarly, *sdhC* belongs to the most reliably predicted targets of IsrR (Supplementary Table S2), however, the membrane protein SdhC was not covered by our proteome analysis. Additional IsrR binding sites were predicted for *sdhA* and *sdhB* (Supplementary Table S2), suggesting that IsrR binds to the RBS of the *sdhA* mRNA and affects its translation. The SdhB protein showed a similar abundance pattern (Figure 5), but was identified by only one peptide and therefore excluded from the general analysis. The effect of IsrR on *sdhB* expression could result from either direct sRNA binding or translational coupling between *sdhA* and *sdhB* (Supplementary Figure S3).

Interestingly, the *in silico* target prediction using CopraRNA2 suggested an even broader effect of IsrR on the expression of TCA cycle genes (Supplementary Table S2). In addition to CitB, SucA, and Sdh, IsrR binding sites were predicted for Mqo and CitZ. Therefore, we compared the stationary phase amount of all TCA cycle proteins between the *isrR*-expressing and non-expressing strains based on their maxLFQ protein levels (Figure 5). Indeed, all proteins encoded by mRNAs with a potential IsrR binding site showed lower abundance in cells containing IsrR, even though the difference in CitZ protein level between HG001 pJLisrR and HG001 pJLctrl was not significant. In contrast, the amounts of CitC, SucCD and FumC were not affected or even higher in the presence of IsrR.

### Expression of IsrR increases sensitivity of *S. aureus* to hydrogen peroxide

**Figure 6:**
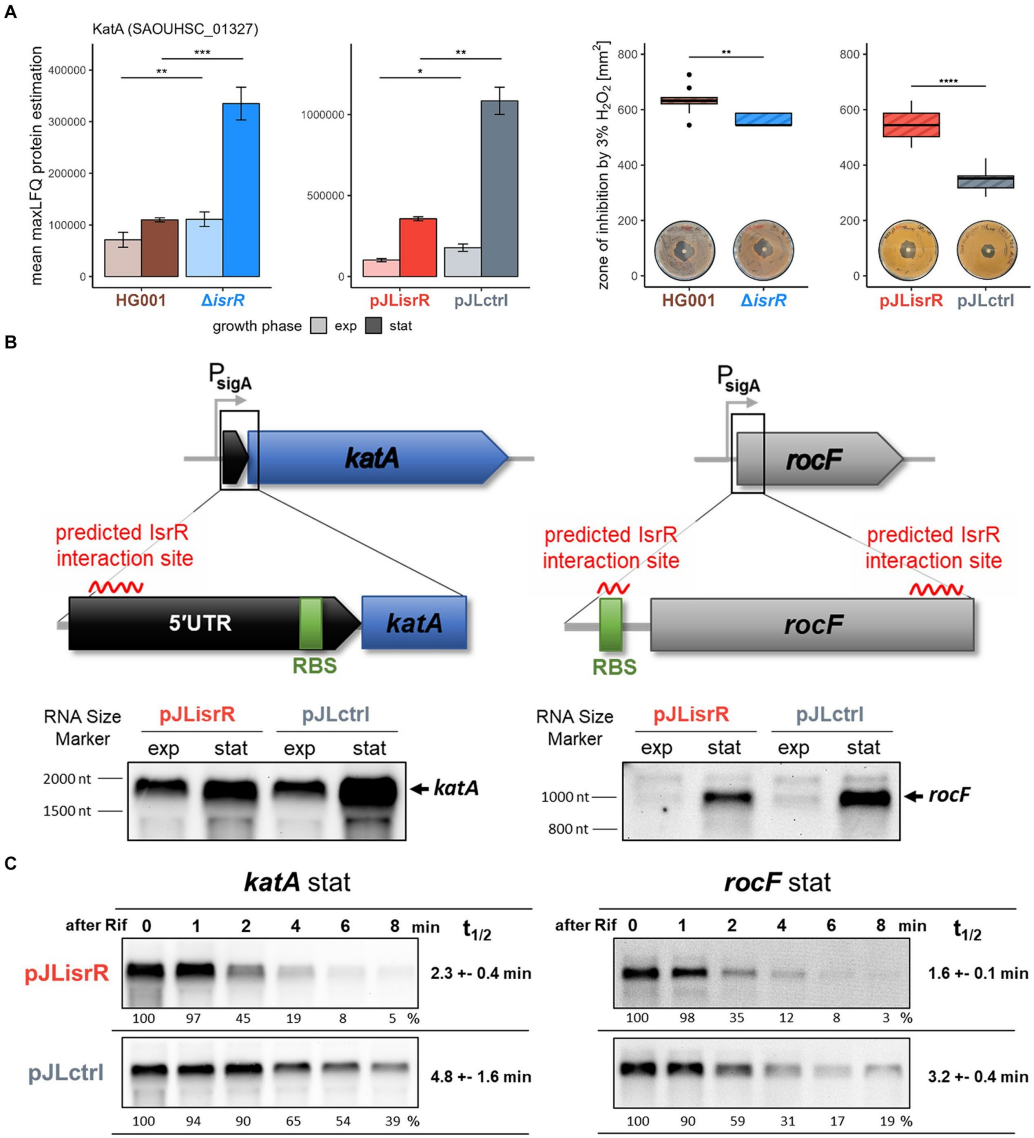
**(A)** Catalase (KatA) protein levels and sensitivity of strains to exogenous hydrogen peroxide (H_2_O_2_). The bar chart shows the amount (mean maxLFQ protein level) of KatA protein in exponential (exp) and stationary (stat) growth phase in *isrR*-expressing (HG001, pJLisrR) and non-expressing strains (∆*isrR*, pJLctrl). Error bars represent the standard deviation of the biological replicates. Statistics: Welch-*t*-test on protein levels (*p* < 0.001***, *p* < 0.01**, *p* < 0.05*). The boxplot depicts the area of inhibition zone to 3% H_2_O_2_ solution determined in four independent disk diffusion assays performed in duplicate. Statistics: Welch-*t*-test (*p* ≤ 0.0001****, *p* ≤ 0.001***, *p* ≤ 0.01**, and *p* ≤ 0.05*). **(B)** Schematic representation of the *katA* and *rocF* genes and their predicted interaction regions with IsrR. CopraRNA2 (Wright et al., 2013) predicted interaction energies: *katA*-IsrR −8.62 kcal/mol at bases −104 to −82 relative to the start codon; *rocF*-IsrR −14.93 kcal/mol at bases 136 to 156 relative to the start codon. Additional IntaRNA2 (Mann et al., 2017) predicted interaction energy for *rocF*-IsrR: −7.48 kcal/mol at bases −14 to −6 relative to the start codon. The effect of IsrR on the mRNA abundance of both genes was examined by Northern blot analysis. **(C)** Influence of IsrR on *katA* and *rocF* mRNA level and stability. The half-lives were determined from two independent experiments.

According to our results, IsrR target mRNAs also encode heme biosynthesis enzymes (HemA and HemY) as well as heme-containing proteins including catalase (KatA) (Table 2). Catalase is an oxidative stress protective protein that detoxifies hydrogen peroxide by converting it into water and oxygen. Its encoding gene is repressed by the peroxide sensor PerR (Horsburgh et al., 2001a). Regulation of *katA* by IsrR is particularly interesting because it was shown earlier that *katA* expression is positively regulated by Fur in *S. aureus*, probably in an indirect manner (Horsburgh et al., 2001b; Cosgrove et al., 2007). KatA is the major enzyme responsible for resistance of *S. aureus* to externally applied hydrogen peroxide (Cosgrove et al., 2007). Since KatA was identified as a very likely IsrR target, we functionally tested whether IsrR also affected hydrogen peroxide sensitivity of *S. aureus*. A disk diffusion assay was performed, where the area of growth inhibition is determined by the capacity of the cells to detoxify exogenous hydrogen peroxide. Consistent with decreased KatA protein abundance in the presence of IsrR (Figure 6A), a significantly larger zone of inhibition was observed in HG001 compared to HG001 Δ*isrR* and in HG001 pJLisrR compared to HG001 pJLctrl (Figure 6A). These results further support our finding that IsrR negatively affects catalase activity by downregulating *katA* expression.

### IsrR promotes increases of protein levels of two very likely targets

The two very likely IsrR targets, SAOUHSC_00304 and SAOUHSC_00827, showed an inverse protein pattern compared to the negatively regulated targets that were discussed so far and can be ascribed to the classical mode of sRNA action. The first positively regulated protein, SAOUHSC_00827, is a low-abundant and largely uncharacterized protein. The second very likely target, SAOUHSC_00304, is the first gene of the *sirTM* operon encoding a sirtuin/macrodomain system most likely involved in the defense against host-derived oxidative stress (Rack et al., 2015). Additionally, Surmann et al. (2014) could show induction of GcvH-L and, to even higher extent, the macrodomain protein SAOUHSC_00306 upon internalization by different mammalian host cells. Interestingly, all proteins encoded by this operon (SAOUHSC_00304, GcvH-L, SAOUHSC_00306, SirTM and LplA2) exhibited the IsrR-dependent abundance pattern (Supplementary Figure S4). The predicted interaction region of IsrR with SAOUHSC_00304 is located upstream of the RBS within the 5’ UTR, as is frequently observed for sRNA-mediated activation of gene expression (Papenfort and Vanderpool, 2015). sRNA binding within the 5’ UTR can prevent the formation of an inhibitory structure in the mRNA thus liberating the RBS or protect the mRNA from degradation by ribonucleases. Indeed, Northern blot analysis using an SAOUHSC_00304 specific probe revealed that the abundance of the 4 kb long *sirTM* operon transcript was considerably higher in the presence of IsrR compared to the respective control strains (Supplementary Figure S4).

### IsrR can affect protein levels by regulating target mRNA stability

In most cases, the predicted interaction region of IsrR with the target mRNA overlaps the RBS sequence (Table 2; Supplementary Table H), implying that reduced protein amounts in the presence of IsrR result from translational repression, which often leads to destabilization of the transcript and thus a decrease in steady-state mRNA levels (Wagner and Romby, 2015). In order to test this possibility, mRNA levels of 10 potential IsrR targets were analyzed by Northern blotting in the same strains and growth conditions as used in the proteome analysis (Supplementary Figure S5). Overall, we observed no or only small effects of IsrR on target mRNA levels, indicating that inhibition of translation does not generally decrease stability of the targeted mRNAs in *S. aureus* as observed, for example, in *B. subtilis* (Gaballa et al., 2008). Only two potential IsrR targets, *katA* and *rocF*, showed clearly reduced mRNA amounts in the presence of IsrR, in both cases the effect was confined to the stationary phase (Figure 6B; Supplementary Figure S5). During exponential growth, CcpA represses the *rocF* gene encoding arginase, whereas the catalase gene *katA* is expressed in both growth phases. However, *katA* mRNA levels were exclusively affected in the stationary phase, which was in agreement with a minor effect of IsrR on KatA protein abundance during exponential growth of *S. aureus* (Figure 6A).

In contrast to most identified IsrR targets, the predicted interaction region within the *katA* mRNA does not overlap the RBS, but is located in the *katA* 5’UTR (Figure 6B; Supplementary Figure S7). IsrR contains three C-rich regions (CRRs) found in different staphylococcal sRNAs (Geissmann et al., 2009), which can pair with the RBS of target mRNAs including the IsrR targets *fdhA* and SAOUHSC_02760 (Coronel-Tellez et al., 2022). The region of IsrR predicted to interact with the *katA* mRNA is located between CRR1 and CRR2 (Supplementary Figure S7). As it seems likely that IsrR exerts its regulatory effect on *katA* through a mechanism other than translation inhibition, we asked if IsrR can directly affect the stability of target mRNAs. We determined the half-lives of selected IsrR targets in *S. aureus* HG001, HG001 pJLctrl and HG001 pJLisrR. For this, cells were grown in TSB and rifampicin was added to exponential and stationary phase cultures, respectively, to prevent transcription initiation. RNA samples removed before and at different time points after rifampicin addition were analyzed by Northern blotting (Figure 6C; Supplementary Figure S6). Strikingly, the mRNA half-lives of *katA* and *rocF* were two- to threefold lower in the *isrR* expressing strain compared with HG001 and the empty vector control strain, whereas the stability of the *citB* mRNA was not affected by IsrR (Table 3). As expected based on *katA* mRNA and protein amounts (Figures 6A,B), the effect of IsrR on *katA* transcript stability was only observed in the stationary phase (Table 3; Supplementary Figure S6). As observed for *citB*, the half-life of the *fdhA* mRNA was equally high in all strains in the stationary phase, however in the exponential phase lower values were measured in HG001 pJLisrR compared with the control strains.

**Table 3:** mRNA half-lives in minutes.

Strain	pJLisrR		pJLctrl		HG001	
Growth phase	exp	stat	exp	stat	exp	stat
*katA*	1.7 +/− 0.2	2.3 +/− 0.4	2.5 +/− 0.4	4.8 +/− 1.6	2.1 +/− 0.1	6.5 +/− 0.9
*rocF*	–	1.6 +/− 0.1	–	3.2 +/− 0.4	–	4.7 +/− 0.5
*fdhA*	0.5 +/− 0.4	3.5 +/− 0.3	1.6 +/− 0.1	3.4 +/− 0.2	1.5 +/− 0.5	4.6 +/− 0.2
*citB*	1.7 +/− 0.4	4.3 +/− 0	1.4 +/− 0.3	2.5 +/− 0.7	1.5 +/− 0.3	3.4 +/− 0

## Discussion

Because iron is an essential nutrient, microbes have evolved multiple strategies to counteract iron limitation. In addition to expressing high-affinity iron uptake systems, they can remodel their metabolism to reduce the need for iron-containing enzymes. Fur, the key regulator of iron homeostasis, primarily acts as a transcriptional repressor of genes encoding iron acquisition systems under iron-replete conditions. However, it was frequently shown that adaptation to iron limitation involves even broader changes in the proteome including downregulation of iron-dependent, non-essential pathways, such as the TCA cycle. This “iron-sparing” response, which enables the bacterial cell to prioritize the utilization of iron, is associated with major changes in cellular metabolism under iron-limiting conditions (Mchugh et al., 2003; Massé et al., 2005; Friedman et al., 2006; Nelson et al., 2019). It is often mediated by Fur-regulated sRNAs, such as RyhB found in many Gram-negative bacteria (Chareyre and Mandin, 2018). RyhB function requires the RNA chaperone Hfq, which generally promotes the interaction between sRNAs and their mRNA targets in Gram-negative bacteria (reviewed in, e.g., Djapgne and Oglesby, 2021). A functional analogous sRNAs, FsrA, was identified in *B. subtilis*, which acts in collaboration with three small, basic proteins, FbpA, B and C (Gaballa et al., 2008; Smaldone et al., 2012b).

Here, we performed the first genome-wide study to characterize the targetome of the Fur-regulated sRNA IsrR of *S. aureus*. We used a mass spectrometry-based approach comparing the proteomes of *S. aureus* strains either lacking or constitutively expressing IsrR. To distinguish direct IsrR targets from indirect IsrR-driven changes of the proteome, experimentally determined candidates were integrated with computationally predicted targets revealed by the CopraRNA tool (Wright et al., 2013), which led to the identification of 21 very likely IsrR targets. The vast majority of these 21 targets (19/21) were negatively affected by IsrR based on the observed protein patterns (Figure 4; Table 2). Of these, 10 proteins are directly related to known functions of Fur-regulated sRNAs, namely Fe-S cluster or heme-containing proteins (CitB, FdhA, MiaB, SAOUHSC_02760, KatA), proteins involved in the TCA cycle and its regulation (CitB, SdhA, SucA, CcpE) and heme biosynthesis (HemA, HemY). These findings are in agreement with other Fur-regulated sRNAs, which mostly target genes encoding enzymes with iron cofactors (Chareyre and Mandin, 2018). Based on the data obtained here, subsequent studies will be carried out to demonstrate the direct interaction between IsrR and the identified targets and to validate the predicted base-pairing regions. For formate dehydrogenase FdhA and SAOUHSC_02760 (COG symbol: GltB2), translational repression by *S. aureus* IsrR was previously demonstrated by Coronel-Tellez et al. (2022). In addition, their study identified nitrate reductase subunit NarG and nitrite reductase subunit NasD as IsrR targets. The *narGHJI* and *nasDEF* operons are induced by the NreC regulator at low oxygen availability (Schlag et al., 2008) and were therefore not expressed under the growth conditions used in our study.

During aerobic growth of *S. aureus* on glycolytic substrates, the TCA cycle is activated in the post-exponential growth phase, catalyzing the oxidation of acetyl-CoA and generation of NADH, which then generates ATP via the respiratory chain. It was shown earlier that the TCA cycle is downregulated during iron-limited growth of *S. aureus* and as a result of Fur deletion, associated with a shift towards fermentation pathways, namely lactate production (Allard et al., 2006; Friedman et al., 2006). Of the TCA cycle, three enzymes were identified as very likely IsrR targets by our combined experimental and computational approach. In addition to the iron-containing aconitase (CitB) and succinate dehydrogenase complex (SdhABC), which are conserved targets of Fur-regulated sRNAs, such as RyhB and FsrA, IsrR is supposed to regulate the 2-oxoglutarate dehydrogenase complex SucAB. In addition, IsrR binding sites were predicted for CitZ (citrate synthase) and Mqo (malate dehydrogenase), and both proteins were present at lower levels in the *isrR*-expressing compared to the non-expressing strains (Figure 5). The regulation of non-iron containing TCA cycle enzymes by iron-responsive sRNAs has been observed before, as in the case of Mqo of *Neisseria meningitidis* (Pannekoek et al., 2017). Interestingly, among the IsrR targets identified by our approach was CcpE, a citrate-binding positive regulator of the aconitase gene (*citB*), which has also been associated with the regulation of virulence genes in *S. aureus* (Hartmann et al., 2013; Ding et al., 2014). Very recently, Barrault et al. (2024) experimentally demonstrated that IsrR interacts with the *citB* and *ccpE* mRNAs and inhibits their translation.

**Figure 7:**
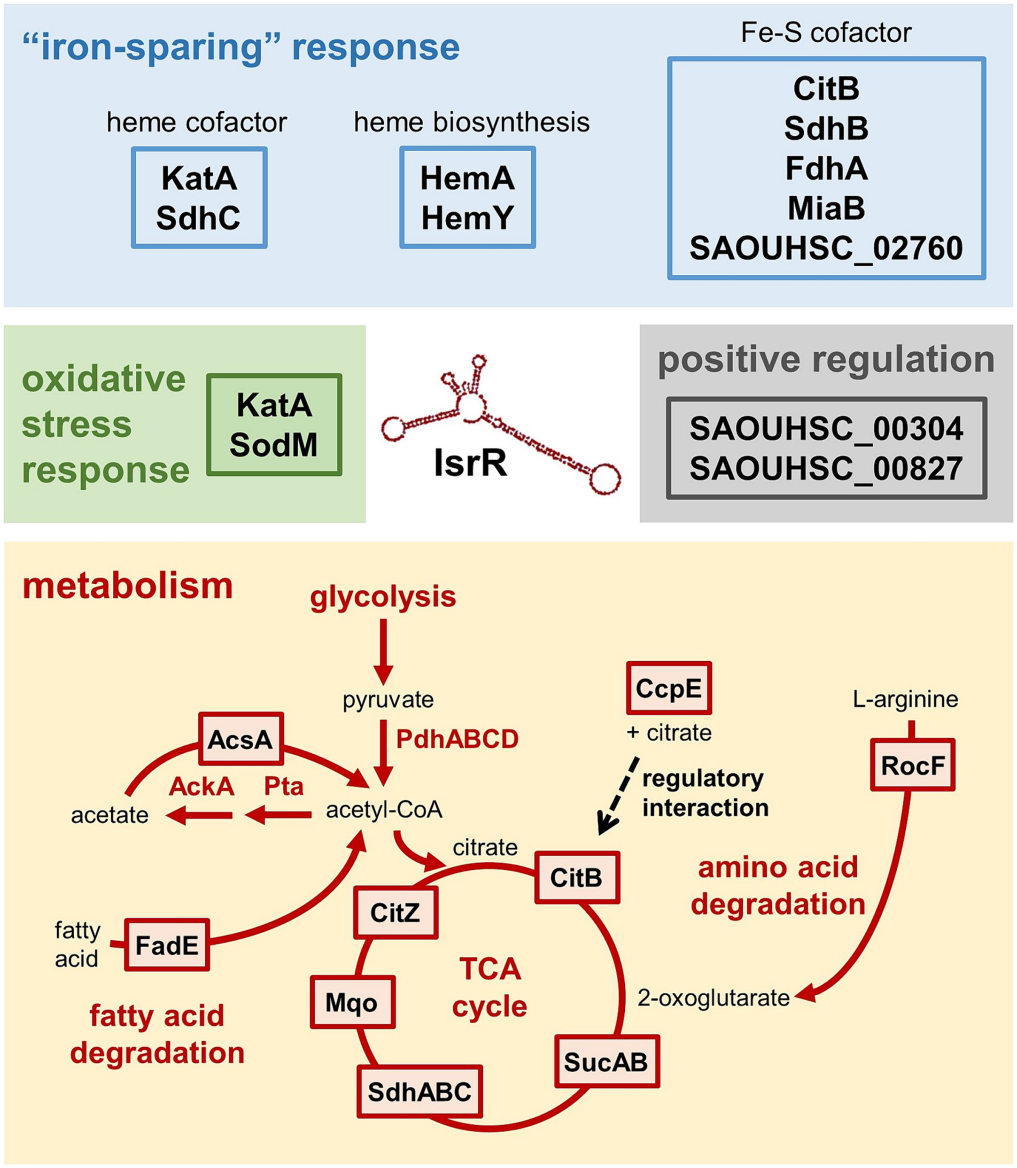
The IsrR targetome of *S. aureus* HG001. Targets identified by the present study are depicted in boxes. In the course of the “iron-sparing” response, cells downregulate iron-consuming, non-essential pathways such as the TCA cycle. This is mediated by IsrR, which decreases the amounts of four proteins involved in the TCA cycle and its regulation (CitB, SdhA, SucA, CcpE) and three proteins (AcsA, RocF, FadE) involved in catabolic pathways generating metabolites that can fuel the TCA cycle. IsrR binding sites were also predicted for Mqo and CitZ, but the changes in protein levels did not meet the significance criterion. IsrR has a negative impact on the oxidative stress response, namely on KatA and SodM. Two proteins were identified as potentially positively regulated by IsrR, SAOUHSC_00827 and SAOUHSC_00304.

In addition to the TCA cycle enzymes, our approach revealed three IsrR targets, AcsA, RocF and FadE, which are involved in catabolic pathways generating metabolites that can fuel the TCA cycle (Figure 7). In the presence of glucose, CcpA represses the TCA cycle as well as the *acsA*, *rocF* and *fadE* genes. Hence, during exponential growth of *S. aureus* in TSB medium, the TCA cycle is not active and the glycolytic end-product pyruvate is converted to acetate, which is secreted into the medium. When glucose is largely consumed, the excreted acetate is taken up and, through acetyl-CoA synthetase (AcsA), converted to acetyl-CoA which is oxidized by the TCA cycle, thereby generating energy for the post-exponential-phase growth (Somerville et al., 2003; Ledala et al., 2014). Ledala et al. (2014) observed reduced acetate catabolism under iron-limiting conditions which might be caused by IsrR-dependent regulation of *acsA* expression. In addition, *S. aureus* grown in TSB with glucose can catabolize amino acids including arginine, proline and histidine in the post-exponential growth phase (Somerville et al., 2002). These amino acids generate glutamate that can enter the TCA cycle after conversion to 2-oxoglutarate via glutamate dehydrogenase GudB (Halsey et al., 2017). Arginase RocF is the first enzyme of the arginine degradation pathway. It is conceivable that downregulation of *rocF* expression by IsrR is related to the repression of the TCA cycle under iron-limiting conditions. In the same context it is interesting to note that another IsrR target protein identified by our analysis, DtpT, is involved in peptide utilization in *S. aureus* (Hiron et al., 2007). Finally, FadE is a long chain-fatty acid-CoA ligase encoded by the *fadXDEBA* operon comprising the genes involved in fatty acid degradation to acetyl-CoA through the canonical β-oxidation pathway (Clark and Cronan, 2005). Kuiack et al. (2023) recently demonstrated that the *fad* operon of *S. aureus* is repressed by glucose and plays a role in metabolizing exogenous palmitic acid.

In contrast to other staphylococci, *S. aureus* contains two SODs, SodA and SodM, metalloenzymes that convert superoxide to hydrogen peroxide (H_2_O_2_) (Clements et al., 1999; Valderas and Hart, 2001). While SodA is present in all staphylococci, SodM is unique to *S. aureus* (Valderas et al., 2002). Both enzymes were assumed to depend on manganese for their activity, however, SodM was found to be cambialistic, possessing equal activity with manganese or iron (Clements et al., 1999; Garcia et al., 2017). In the present study, we noticed that SodM was a potential IsrR target based on the proteomic data of both experimental approaches (Supplementary Table S1). However, no IsrR interaction site was predicted using CopraRNA2. In order to investigate if *S. aureus* SODs exhibit differential sRNA-dependent regulation as described for SodA and SodB of *E. coli* (Massé and Gottesman, 2002), we calculated the IsrR-*sodM* interaction using IntaRNA2 (Mann et al., 2017). This revealed that indeed, there is a putative interaction site located in the RBS of the *sodM* transcript with a predicted interaction energy of −7.99 kcal/mol. The interaction energy for the CopraRNA2-based *in silico* prediction was in the range of −2.35 kcal/mol to −212.21 kcal/mol with a median of −13.35 kcal/mol. The CopraRNA2 approach exploits prediction of conserved targets in closely related organisms to gain specificity in prediction, which means, however, that organism-specific targets like *sodM* elude detection.

Moreover, IsrR clearly has a greater impact on the oxidative stress response, which is in line with the close connection of oxidative stress resistance and metal ion homeostasis. The same holds true for other iron-responsive sRNAs, such as RyhB of *E. coli* that regulates SodB and the methionine sulfoxide reductase MsrB (Bos et al., 2013). In addition to *sodM*, our study revealed that IsrR targets *katA* mRNA and thus contributes to the regulation of cellular catalase levels. Catalase is a heme-dependent enzyme that catalyzes the conversion of H_2_O_2_ into water and oxygen, thus fulfilling an important function in preventing the formation of toxic hydroxyl radicals in the course of the Fenton reaction. *S. aureus* possesses one catalase (KatA) and the alkyl hydroperoxide reductase AhpCF that reacts with H_2_O_2_ and organic hydroperoxides. The *katA* and *ahpCF* genes are repressed by the peroxide sensor PerR (Horsburgh et al., 2001a). In addition, it was shown earlier that *katA* expression is positively regulated by Fur, probably in an indirect manner, which interestingly led the authors to speculate on the regulation by an iron-responsive sRNA (Horsburgh et al., 2001b; Cosgrove et al., 2007). The catalase KatA is the major enzyme conferring H_2_O_2_ resistance to *S. aureus* (Gaupp et al., 2012). By performing H_2_O_2_ plate assays we have demonstrated here that strains expressing IsrR and, as a result, possessing lower KatA protein levels, show clearly increased H_2_O_2_ sensitivity. Horsburgh et al. (2001b) observed an impaired growth phenotype of the *fur* mutant in rich medium, which was partially suppressed by manganese, thereby suggesting that the lack of Fur leads to reduced oxidative stress resistance (Aguirre and Culotta, 2012).

In the canonical mechanism of sRNA action, i.e., inhibition of translation, the sRNA targets the RBS of its target mRNAs. In addition, base pairing with the 5’UTR of the mRNA can promote translation by disrupting secondary structures that would otherwise block the access of ribosomes. Translational repression often leads to transcript destabilization, and hence a decrease in the target mRNA amount (reviewed in Wagner and Romby, 2015). In *S. aureus*, sRNA-mRNA duplexes can be degraded by the double-strand specific endonuclease RNase III (Romilly et al., 2012). In addition, sRNA base-paring with the RBS can affect target mRNA stability through exposure of ribonuclease cleavage sites in the absence of bound ribosomes; it was, e.g., demonstrated that RNase Y, the functional equivalent of RNase E in Gram-positive bacteria, cleaves the *ppnKB* mRNA following translational repression by the RoxS sRNA in *B. subtilis* (Durand et al., 2015). In most cases, IsrR interacts with the target mRNAs at the RBS, suggesting that it acts at the level of translation. However, for most IsrR targets we observed no or only small effects of IsrR on mRNA levels as already suggested for *fdhA* and SAOUHSC_02760 by Coronel-Tellez et al. (2022). It should be noted that protein levels of downstream genes encoded on the same transcript can be affected without transcript degradation by a process known as translational coupling. Ribosomes can re-initiate translation when the start codon of the downstream gene is located within a certain distance to the stop codon of the upstream gene (e.g., Adhin and Van Duin, 1990; Levin-Karp et al., 2013; Osterman et al., 2013; Huber et al., 2023). This can be illustrated by the example of the *sucAB* operon with an intergenic distance of 13 nucleotides, whose transcript levels were not affected by IsrR (Supplementary Figure S5). Although only a single IsrR binding site located in the RBS of *sucA* was predicted, both proteins, SucA and SucB, exhibit the typical pattern of IsrR-dependent regulation (Figure 5).

In contrast to the majority of target mRNAs, in the case of *katA* the IsrR-mRNA interaction is predicted upstream of the RBS (Supplementary Figure S7B). In addition, we observed a strong effect of IsrR expression on *katA* mRNA levels, which led us to investigate if binding of IsrR can directly trigger degradation of target mRNAs (Pfeiffer et al., 2009; reviewed in, e.g., Laalami et al., 2014). Half-life measurements of specific mRNAs after rifampicin-mediated inhibition of transcription demonstrated that the *katA* mRNA was more rapidly degraded in *S. aureus* strains expressing *isrR*. The IsrR base-pairing region is located in a predicted stem-loop structure in the 5’UTR of *katA* (Supplementary Figure S7B), so that binding of IsrR potentially leads to disruption of the stem-loop resulting in decreased stability of the mRNA. Taken together, these findings suggest that IsrR, in addition to inhibiting translation initiation, can downregulate target protein levels by affecting mRNA stability. In the case of *rocF*, which also exhibited reduced transcript stability in the presence of IsrR, CopraRNA analysis predicted an IsrR interaction site within the CDS; however, a second IsrR binding sequence can be found in *rocF* according to IntaRNA2 (Mann et al., 2017) that overlaps the RBS (Figure 6B; Supplementary Figure S7C). Therefore, IsrR might inhibit translation of the *rocF* mRNA and subsequently affect mRNA stability. In line with this, similar interaction sites in the *rocF* mRNA were reported previously (Rochat et al., 2018) for another staphylococcal sRNA, RsaE, which is involved in the adaptation to anaerobic conditions (Geissmann et al., 2009; Bohn et al., 2010). *S. aureus* carbon metabolism, and in particular the activity of the TCA cycle, is determined by the availability of iron and oxygen (Ledala et al., 2014). Interestingly, IsrR and RsaE share additional targets, namely mRNAs encoding TCA cycle enzymes including CitB, suggesting a functional connection between IsrR and RsaE. The present study revealed that the Fur-regulated sRNA IsrR affects multiple metabolic pathways connected to the TCA cycle as well as the oxidative stress response of *S. aureus* (Figure 7).

## Data availability statement

The datasets presented in this study can be found in online repositories. The names of the repository/repositories and accession number(s) can be found in the article/Supplementary material.

## Author contributions

AG: Conceptualization, Data curation, Formal analysis, Investigation, Visualization, Writing – original draft, Writing – review & editing. LB: Conceptualization, Data curation, Formal analysis, Investigation, Visualization, Writing – original draft, Writing – review & editing. CH: Data curation, Investigation, Methodology, Writing – review & editing. AR: Investigation, Methodology, Writing – review & editing. SM: Data curation, Formal analysis, Methodology, Writing – review & editing. KS: Methodology, Supervision, Writing – review & editing. UV: Conceptualization, Funding acquisition, Supervision, Writing – review & editing. UM: Conceptualization, Formal analysis, Methodology, Supervision, Visualization, Writing – original draft, Writing – review & editing.
