# Supplementary material for: Exploring the targetome of IsrR, an iron-regulated sRNA controlling the synthesis of iron-containing proteins in Staphylococcus aureus
Source: Front Microbiol. 2024 Jul 5;15:1439352. doi: 10.3389/fmicb.2024.1439352 (PMC11257911; doi:10.3389/fmicb.2024.1439352)
Supplement: Supplementary file 1 [file Data_Sheet_1.docx]

Supplemental Data for

Exploring the targetome of IsrR, an iron-regulated sRNA controlling the synthesis of iron-containing proteins in *Staphylococcus aureu*s

A. Ganske, L. M. Busch, C. Hentschker, A. Reder, S. Michalik, K. Surmann, U. Völker, U. Mäder

University Medicine Greifswald, Interfaculty Institute for Genetics and Functional Genomics, Greifswald, Germany

# Supplemental Data Index

[Supplemental Data Index 1](#_Toc170132415)

[1 Construction of isogenic *S. aureus* HG001 mutants using pBASE6 system. 2](#_Toc170132416)

[2 Supplementary Tables 3](#_Toc170132417)

[Table A. Oligonucleotides used for preparation of Northern blot probes. 3](#_Toc170132418)

[Table B. Oligonucleotides used for plasmid constructions. 4](#_Toc170132419)

[Table C. Reversed phase liquid chromatography (RPLC). 5](#_Toc170132420)

[Table D. Mass spectrometry. 5](#_Toc170132421)

[Table E. Spectronaut^TM^ parameters used for data analysis of mass spectrometry data. 6](#_Toc170132422)

[Table F. R packages. 7](#_Toc170132423)

[Table G. Organism sets for IsrR targets *in silico* prediction. 8](#_Toc170132424)

[Table H. IsrR interaction sites on the respective mRNAs of target candidates ………….. 11](#_Toc170132425)

[3 Supplementary Figures 13](#_Toc170132426)

[Figure S1. Iron-related growth of *S. aureus* strains and expression of *isrR*. 14](#_Toc170132427)

[Figure S2. Aconitase activity determined as absorbance units per minute and normalized to the respective protein concentration. 15](#_Toc170132428)

[Figure S3. Schematic representation of the *S. aureus* operon organization of *sdhCAB* and *citB* and their predicted interaction region with 15](#_Toc170132429)

[Figure S4. Protein levels and regulatory effects of IsrR on the *sirTM* Operon. 16](#_Toc170132430)

[Figure S5. The mRNA levels of ten identified IsrR targets negatively affected by presence of the sRNA were determined by Northern blot analysis. 17](#_Toc170132431)

[Figure S6. Level and stability of selected experimentally identified mRNAs from IsrR targets. …………………………………………………………………………………………………………………….18](#_Toc170132432)

[Figure S7. Secondary structure models 20](#_Toc170132433)

[4 References 21](#_Toc170132434)

# Construction of isogenic *S. aureus* HG001 mutants using pBASE6 system.

The shuttle vector pBASE6 (Geiger et al. 2012) is a derivative of the vector pBT2 (Brückner 1997) and carries an additional *secY* antisense RNA from pKOR1 (Bae und Schneewind 2006). The plasmid pBT2 was constructed as described in Brückner (1997) and carries the beta-lactamase gene (*bla*) for resistance to beta-lactam antibiotics, and the ColE1 origin for replication in *E. coli* from plasmid pBR322 (Bolivar et al. 1992). This enables selection of *E. coli* clones carrying the plasmid due to their ampicillin resistance. *E. coli* strains were grown aerobically in lysogeny broth (LB) media with 100 µg/ml ampicillin at 37 °C while shaking vigorously. For replication and chromosomal integration in *S. aureus*, pBT2 contains the temperature-sensitive pE194Ts replicon and the chloramphenicol acetyltransferase gene (*cat*_194_) for selection of plasmid-containing *S. aureus* clones. The *secY* asRNA is inducibly expressed to repress the synthesis of the essential protein SecY (Bae und Schneewind 2006), enabling selection for loss of the chromosomally integrated plasmid. This results in a mutant that only carries the antibiotic resistance cassette, devoid of any heterologous DNA.

To perform gene replacement, we used two 1000 bp fragments flanking the *isrR* locus and the erythromycin resistant cassette *ermB* (Thakker-Varia et al. 1985). These fragments were cloned using the In-Fusion® HD Cloning Kit from Takara Bio into the linearized vector pBASE6 via the SLIC (Sequence and Ligase Independent Cloning) method (Li und Elledge 2012) to generate the Δ*isrR*::*ermB* mutagenesis vector pBDisrR in *E. coli* DC10B. After verifying the resulting plasmid, it was used for transformation of *S. aureus* RN4220. Subsequently, the passaged mutagenesis plasmid was introduced into the *S. aureus* HG001 strain through transformation. The mutagenesis was performed as previously described (Bae und Schneewind 2006) to replace the entire *isrR* sequence with the corresponding resistance cassette. The resulting *isrR* gene replacement mutant strain SGB007 was verified by PCR, Northern Blot, and DNA sequence analysis. The same mutagenesis procedure was used to construct the chromosomal *fur* mutant SGB009 and the double mutant SGB010.

# Supplementary Tables

## Table A. Oligonucleotides used for preparation of Northern blot probes.

| **probe** | **oligonucleotide** | **Sequence^a^** |
| --- | --- | --- |
| IsrR/S596 | SA_S596_for | TCACTAATGTATAATAGTAGTTG |
|  | SA_S596_revT7 | GAAATTAATACGACTCACTATAGGGAGAAGTGTCGTAAGGGTTTACTG |
| citB | SA_citB_for | ATGGCTGCAAATTTTAAAGAG |
|  | SA_citB_revT7 | GAAATTAATACGACTCACTATAGGGAGATTTCACCATCTACATCACGA |
| sucA | SA_sucA_for | ATGACTAACGAAAGAAAAGAAG |
|  | SA_sucA_revT7 | GAAATTAATACGACTCACTATAGGGAGACAATTGAAAAACGCTTTGC |
| katA | SA_katA_for | ATGCAAGATATTTACTTTTTAGAG |
|  | SA_katA_revT7 | GAAATTAATACGACTCACTATAGGGAGACACGATCTGTAGCTATAATTTC |
| sdhA | SA_sdhA_for | CAGAGAAACATCTTATTGTTGTC |
|  | SA_sdhA_revT7 | GAAATTAATACGACTCACTATAGGGAGACATTGAGTTTGTTGTTTTACCG |
| acsA | SA_acsA_for | GAAAGTCGAAGTTTATAAAGG |
|  | SA_acsA_revT7 | GAAATTAATACGACTCACTATAGGGAGAGTATAATGTAAAATCAAACCATC |
| fdhA | SA_fdhA_for | GCAAGAACATTTGGTGGTTAC |
|  | SA_fdhA_revT7 | GAAATTAATACGACTCACTATAGGGAGACATGGCATTACATGGACATAC |
| rocF | SA_rocF_for | GACAAAGACAAAAGCAATTG |
|  | SA_rocF_revT7 | GAAATTAATACGACTCACTATAGGGAGACTATTGTATTTTCAATTACTTCCT |
| miaB | SA_miaB_for | TGAACGAAGAACAAAGAAAAGC |
|  | SA_miaB_revT7 | GAAATTAATACGACTCACTATAGGGAGACGTCTCCTTCTTTAGACCATAC |
| SAOUHSC_02760 | SA_02760_for | GACGTTTCTTACAGTCATGC |
|  | SA_02760_revT7 | GAAATTAATACGACTCACTATAGGGAGACGCTTTAGCTAGACCTTTAG |
| hemA | SA_hemA_for | GAGATGATGHCCTTACGAATTGC |
|  | SA_hemA_revT7 | GAAATTAATACGACTCACTATAGGGAGAACACTTACA |
| SAOUHSC_00304 | SA_00304_for | GGTTAAATTAAGCGTATTAGACTATG |
|  | SA_00304_revT7 | GAAATTAATACGACTCACTATAGGGAGAGTTATCCTTCGCTGTATGTATC |

^a^ The sequence GAAATTAATACGACTCACTATAGGGAGA[N]_x_ corresponds to the T7 promoter.

## Table B. Oligonucleotides used for plasmid constructions.

| **SGB007 mutant generation** | | | |
| --- | --- | --- | --- |
| **N°** | | **Name** | **Sequence** |
| 1 | DS596_up_for_ex.pBASE6 | | AGCTCGGTACCCGGGCCTTTTGTGGCTGACGACG |
| 2 | D596_up_rev | | CATGGTTGAAATCCTAAACTACTATTATACATTAG |
| 3 | D596_do_for | | ATTCAACGGACGTAAATAAATAAAAGCAGTAAACCCTTAC |
| 4 | DS596_do_rev_ex.pBASE6 | | AGATCTGCGCGCTAGGATCAAATGCGATTTATGAG |
| 5 | pBASE6_linear_for | | CTAGCGCGCAGATCTGTCGA |
| 6 | pBASE6_linear_rev | | CCCGGGTACCGAGCTCCGGA |
| 7 | pBASE6_ctrl_for | | CAGGAGAGCGTTCACCGACA |
| 8 | pBASE6_ctrl_rev | | GTGATACCCCTCTTTCCATG |
| 9 | S596_INFUSION_for | | GGAGGATGATTATTTTCACTAATGTATAATAGTAGTTGAA |
| 10 | S596_INFUSION_rev | | TTATGCATTAGAATAAGTGTCGTAAGGGTTTACTG |
| 11 | DS596_Ins.ctrl_do_for | | TGCGCCATTTACCGTCA |
| 12 | DS596_Ins.ctrl_do_rev | | AGCATTATTCGCGAAAACTTAGA |
| 13 | ermB_+SD_for | | AGGATTTCAACCATGAATAAAAAC |
| 14 | ermB_just_for | | ATGAATAAAAACATTAAATACTCAC |
| 15 | ermB_just_rev | | TTATTTACGTCCGTTGAATAATAAG |
| 16 | ermB_screen_rev | | CCGATTTCGTATACTGTGTC |
| **SGB009 mutant generation** | | | |
| 17 | DFur_up.for_pBASE6.ex | | TTTTATTTGATGCCTCAAGACATTTTTAGTCATGTGTATTG |
| 18 | DFur_up.rev_ermC.ex | | ATGCGATTATTGAATAAAACGTAGGTTAAATTAACCTTCG |
| 19 | DFur_do.for_ermC.ex | | ATTATGTCTTTTGCGCAGAACAATAAAATGCAAGCTTTTCTC |
| 20 | DFur_do.rev_pBASE6.ex | | TAATTTTTTTAAGGCAGTTCGCTTATTGTTTCGTCTAATG |
| 21 | pJL_ermC_for | | TTTTATTCAATAATCGCATCC |
| 22 | pJL_ermC_rev | | TTCTGCGCAAAAGACATAATCG |
| 23 | pBASE6_linear_for2 | | AACTGCCTTAAAAAAATTAC |
| 24 | pBASE6_linear_rev2 | | TTGAGGCATCAAATAAAACG |
| 25 | DFur_Ins.Ctrl_for | | TTATCAGTTGTTAATTGAGCAAC |
| 26 | DFur_Ins.Ctrl_rev | | CACCCAATTCATCATGAGAATA |
| **pJLisrR plasmid generation** | | | |
| 27 | pJL_linear_for | | TATTCTAATGCATAATAAATACTG |
| 28 | pJL_linear_INFUSION_rev | | GATAATCATTTTCAACGTTAATTATAACTAATTAAAAATG |
| 29 | IsrR_INFUSION_rev | | TTATGCATTAGAATAAAAAAAACAAAAGCAGTAAACC |
| 30 | IsrR_INFUSION_for | | GTTGAAAATGATTATCAATACC |
| 31 | pJL_screen_for | | GACTAAACCAAATGCTAACC |
| 32 | pJL_screen_rev | | GTCACTTTGCTTGATATATGAG |
| **pJLctrl plasmid generation** | | | |
| 33 | pJL_ctrl_FragA_for | | GGAGGATGATTATTTTATTCTAATGCATAATAAATACTG |
| 34 | pJL_ctrl_FragA_rev | | TAGTGACATTAGAAAACCGACTG |
| 35 | pJL_ctrl_FragB_for | | TTTCTAATGTCACTACCCTCG |

## Table C. Reversed phase liquid chromatography (RPLC).

| Instrument | Ultimate 3000 RSLC (Thermo Scientific) |
| --- | --- |
| Trap column | 75 μm inner diameter, packed with 3 μm C18 particles (Acclaim PepMap100, Thermo Scientific) |
| Analytical column | Accucore 150-C18, (Thermo Fisher Scientific)  25 cm x 75 μm, 2.6 μm C18 particles, 150 Å pore size |
| Buffer system | binary buffer system consisting of 0.1% acetic acid in HPLC-grade water (buffer A) and 100% ACN in 0.1% acetic acid (buffer B) |
| Flow rate | 300 nl/min |
| Gradient | linear gradient of buffer B from 2% up to 25% |
| Gradient duration | 120 min |
| Column oven temperature | 40 °C |

## Table D. Mass spectrometry.

| **Instrument** | **Q Exactive HF mass spectrometer** |
| --- | --- |
| Electrospray | Nanospray Flex Ion Source |
| Operation mode | data-independent |
| **Full MS** |  |
| MS scan resolution | 60000 |
| Norm. AGC target | 5e6 |
| maximum ion injection time for the MS scan | 200 ms |
| Scan range | 333 to 1650 m/z |
| RF Lens | 50% |
| Spectra data type | profile |
| **dd-MS2** |  |
| Precursor mass range | 333 to 1650 m/z |
| Resolution | 30,000 |
| Norm. MS/MS AGC target | 3e6 |
| Maximum ion injection time mode | auto |
| Spectra data type | profile |
| Microscans | 1 |
| Isolation window | 56 windows, 13 m/z, 2 m/z overlap |
| Define first mass | 200 |
| Dissociation mode | higher energy collisional dissociation (HCD) |
| Normalized collision energy | 27.5 % |

## Table E. Spectronaut^TM^ parameters used for data analysis of mass spectrometry data.

| Parameter | Setting |
| --- | --- |
| Identification  Pvalue Estimator  Precursor Qvalue Cutoff | Kernel Density Estimator  0.001 |
| Quantification  Precursor Filtering  Imputation Strategy  Quantity MS Level  Quantity Type  Cross-Run Normalization  Normalization Strategy  Row Selection | Identified (Qvalue)  Use Background Signal  MS2  Area  True  Global Normalization (Median)  Identified in at least 1 Run (Sparse) |

## Table F. R packages.

| **Package** | **Version** | **Reference** |
| --- | --- | --- |
| FactoMineR | 2.4 | Lê et al. 2008 |
| ggpattern | 1.0.1 | Mike FC et al. 2022 |
| ggpubr | 0.4.0 | Kassambara 2020 |
| ggrepel | 0.9.1 | Slowikowski 2021 |
| ggtext | 0.1.2 | Wilke und Wiernik |
| Hmsic | 4.7-1 | Harrell Jr 2022 |
| iq | 1.9.6 | Pham et al. 2020 |
| openxlsx | 4.2.5 | Schauberger und Walker 2021 |
| patchwork | 1.1.1 | Pedersen 2020 |
| PECA | 1.30.0 | Suomi et al. 2021 |
| readr | 2.1.2 | Wickham et al. 2022 |
| readxl | 1.4.0 | Wickham und Bryan 2022 |
| rstatix | 0.7.0 | Kassambara 2021 |
| scales | 1.2.0 | Wickham und Seidel 2022 |
| tidyverse | 1.3.1 | Wickham et al. 2019 |
| vroom | 1.5.7 | Hester et al. 2021 |

## Table G. Organism sets for IsrR targets *in silico* prediction.

| **Organism** | **Accession** | **IsrR sequence** | **Set** **≥ 85% identity** | **Set  ≥ 85% identity + AureoWiki** | **Set  ≥ 90% coverage** | **Set  ≥ 90% coverage + AureoWiki** |
| --- | --- | --- | --- | --- | --- | --- |
| *S. aureus* NCTC8325 | NC_007795 | GTTGAAAATGATTATCAATACCACATAGAACATCCCCCCCACAACGTTTCGTTCTTGTTGGATTGGTCATTTTCAAATATTCCCCTTTTATATGCCCGTAAAAGACAATATACGTTATAACAACGTTTTATAAAAGCAGTAAACCCTTACGACACTTTAGGTTTACTGCTTTTGTTTTTTT | X | X | X | X |
| *S. aureus* COL | NC_002951 | GTTGAAAATGATTATCAATACCACATAGAACATCCCCCCCCACAACGTTTCGTTCTTGTTGGATTGGTCATTTTCAAATATTCCCCTTTTATATGCCCGTAAAAGACAATATACGTTATAACAACGTTTTATAAAAGCAGTAAACCCTTACGACACTTTAGGTTTACTGCTTTTGTTTTTTT |  | X |  | X |
| *S. aureus* N315 | NC_002745 | GTTGAAAATGATTATCAATACCACATAGAACATCCCCCCCACAACGTTTCGTTCTTGTTGGATTGGTCATTTTCAAATATTCCCCTTTTATATGCCCGTAAAAGACAATATACGTTATAACAACGTTTTATAAAAGCAGTAAACCCTTACGACACTTTAGGTTTACTGCTTTTGTTTTTTT |  | X |  | X |
| *S. aureus* Newman | NZ_CP023390 | GTTGAAAATGATTATCAATACCACATAGAACATCCCCCCCACAACGTTTCGTTCTTGTTGGATTGGTCATTTTCAAATATTCCCCTTTTATATGCCCGTAAAAGACAATATACGTTATAACAACGTTTTATAAAAGCAGTAAACCCTTACGACACTTTAGGTTTACTGCTTTTGTTTTTTT |  | X |  | X |
| *S. aureus* USA300_FPR3757 | NC_007793 | GTTGAAAATGATTATCAATACCATATAGAACATCCCCCCCACAACGTTTCGTTCTTGTTGGATTGGTCATTTTCAAATATTCCCCTTTTATATGCCCGTAAAAGACAATATACGTTATAACAACGTTTTATAAAAGCAGTAAACCCTTACGACACTTTAGGTTTACTGCTTTTGTTTTTT |  | X |  | X |
| *S. argenteus* | NC_016941 | GTTGAAAATGATTATCAATACCACATAGAACATCCCCCCCACAACGTTTCGTTCATGTTGGATTGGTCATTTTCAGATATTCCCCTTTTATATGCCCGTAAAAGACAATATACGTTATAACAACGTTTTATAAAAGCAGTAAACCCTTACGACACTTTAGGTTTACTGCTTTTGTT | X | X | X | X |
| *S. caprae* | NZ_AP018587 | AAAAGCAGTAAACCTCAAGTGTCAAAGGTCTACTGCTTTTATTGAAACGTTGTTTCTCAACGTTAGTCTTTTACGGGCATATAAAAGGGGATAATTGAAAATGACCAATCCAATAAGAACGAAATATATGTGGGGGGGAATGTTCTAGTCGGTATTGATAATCATTTTCAAC | X | X | X | X |
| *S. condimenti* | NZ_CP018776 | TTTACGGGCATATATAAAAGGGGATATGAAAATGACCAATACTATAAGAACGAAATTGTGGGGGGATGTCTCATTTGGTATTGATAATCATTTTCAA | X | X |  |  |
| *S. debuckii* | NZ_CP033460 | TTGAAAATGATTATCAATACCAAATGAGACATCCCCCCACATTTTCGTTCTTATTGTATTGGTCATTTTCATATCCCCTTTTATATATGCCCGTAAA | X | X |  |  |
| *S. epidermidis* | NZ_CP035288 | AAAAACAGCAGACCTCAAGTGTCAAAGGTCTACTGCTTTTATTGAAACGTCATTTCTTAACGTTAGTCTTTTACGGGCATATAAAAGGGGATAGTTGAAAATGACCAATCCAAAAGAACAACTTGTATGTGGGGGGAATGTTTTTCAATTGGTATTGATAATCATTTTCAA |  |  | X | X |
| *S. equorum* | NZ_CP066013 | GTCTTTTACGGGCATATAAAAGGGGATATTTCTGAAAATGATCAATCCAATAAGATCGAAATTCGTGTGGGGGGGAATGTCTTATGTTTGGAATTGATAATCATTTTCAA | X | X |  |  |
| *S. haemolyticus* | NZ_CP013911 | TTGAAAATGATTATCAATACCGAATAAGACAATTCCCCCCACATATATTTCGTTCTTAAAGGATTGGTCATTTTCAAGTTATTCCCCTTTTATATGCCCGTAAAAGAC | X | X |  |  |
| *S. kloosii* | NZ_CP027846 | GTCTTTTACGGGCATATAAAAGGGGATATTTCTGAAAATGATCATTCCATCAAAGAACGAAATGTGTGGGGGGAATGTCTTTTCGGAATTGATAATCATTTTCAA | X | X |  |  |
| *S. lloydii* | NZ_CP064056 | GTCTTTTACGGGCATATAAAAGGGGATATTTCTGAAAATGATCATTCCATCAAAGAACGAAATGTGTGGGGGGAATGTCTTTTTGGAATTGATAATCATTTTCAA | X | X |  |  |
| *S. lugdunensis* | NZ_CP014022 | TTGAAAATGATTATCAATACCAAATAGAACTCCCCCCACATATTCGTTCTTATGGATTGATCATTTTCGAATTCCCCTTTTATATGCCCTTAAAAGAC | X | X |  |  |
| *S. pasteuri* | NZ_CP067014 | AAAAAAAGTAGTAAACCTCAAGTGTCTAAAGGTTTACTACTTTATTTGAACGTCGTTTCCAACGTTAGTCTTTTACGGGCATATAAAAGGGATTATGAAAATGACCAATACATAAGAACGAAATATATGTGGGGGGAAAGTTCTTTTTGGTATTGATAATCATTTTCAA |  |  | X | X |

| *S. pseudoxylosus* | NZ_CP075500 | TTGAAAATGATTATCAATTCCAAAAGTAAGACATTCCCCCCCCACACATATTTCGTTCTTATTTGGATTGATCATTTTCAAAAATATCCCCTTTTATATGCCCGTAAAAGAC | X | X |  |  |
| --- | --- | --- | --- | --- | --- | --- |
| *S. saccharolyticus* | NZ_CP068029 | AAAAGCAGTGGACCTCAAGTGTCAAAGGTCTACTACTTTTATTGAAACGTTGTTTCTCAACGTTAGTCTTTTACGGGCATATAAAAGGGGATAATTGAAAATGACCAATCCAATAAAACGTCATGTATGTGGGGGGAATGTTCTAGTTGGAATTGATAATCATTTTCAA |  |  | X | X |
| *S. saprophyticus* | NZ_CP031196 | GTCTTTTACGGGCATATAAAAGGGGATATTTTTGAAAATGAACAATCCAAATAAGAACGAAATATGTGGGGGGGAATGTCTTATTTTTGGTATTGATAATCATTTTCAA | X | X |  |  |
| *S. schweitzeri* | NZ_LR134304 | GTTGAAAATGATTATCAATACCACATAGAACATCCCCCCCACAACGTTTCGTTCATGTTGGATTGGTCATTTTCAGATATTCCCCTTTTATATGCCCGTAAAAGACAATATACGTTATAACAACGTTTTATAAAAGCAGTAAACCCTTACGACACTTTAGGTTTACTGCTTTTGTT | X | X | X | X |
| *S. simiae* | NZ_LT906460 | TTGAAAATGATTATCAATACCAAATAGAACATTCCCCCCCCACATACGTTTCGTTCAAAATTGGATTGGTCATTTTCAGATATTCCCCTTTTATATGCCCGTAAAAGACTATATACGTTATAACAACGTTTTTATAAAAGCAGTAAACCTTATGACACTTTAGGTTTACTGCTTTTATT | X | X | X | X |
| *S. warneri* | NZ_CP032159 | AAAAGCAGTAAACCTCAAGTGTCTAAAGGTCTACTGCTTTATTAAAACGTTTTTCAACGTTAGTCTTTTACGGGCATATAAAAGGGGATTATATGAAAATGACCAATCCTTAAGAACGAAATATATGTGGGGGGGGAAAGTTCTTTTTGGTATTGATAATCATTTTCAAC | X | X | X | X |
| *S. xylosus* | NZ_CP008724 | GTCTTTTACGGGCATATAAAAGGGGATATTTTTGAAAATGATCAATCCAATTAAGAACGAAATATGTGTGGGGGGGAATGTCTTAAGTTTGGAATTGATAATCATTTTCAA | X | X |  |  |

## Table H. IsrR interaction sites on the respective mRNAs of target candidates.

| **Protein** | **locus tag** | **Interaction energy [kcal/mol]** | **Interaction start site relative to start codon** | **Interaction stop site relative to start codon** | **RBS interaction** | **Experimentally determined interaction site according to Coronel-Tellez *et al.* (2022)** |
| --- | --- | --- | --- | --- | --- | --- |
| FadE | SAOUHSC_00198 | -12.58 | -38 | 18 | likely |  |
|  | SAOUHSC_00304 | -16.86 | -83 | -63 | not likely |  |
| CcpE | SAOUHSC_00679 | -13.65 | -11 | -5 | likely |  |
| DtpT | SAOUHSC_00738 | -13.26 | -34 | 9 | likely |  |
|  | SAOUHSC_00827 | -13.66 | 230 | 254 | not likely |  |
|  | SAOUHSC_00875 | -13.47 | -21 | -9 | likely |  |
| SdhA | SAOUHSC_01104 | -12.21 | -17 | 33 | likely |  |
| MiaB | SAOUHSC_01269 | -19.97 | -27 | 42 | likely |  |
| KatA | SAOUHSC_01327 | -8.62 | -104 | -82 | not likely |  |
| CitB | SAOUHSC_01347 | -16.09 | -38 | 6 | likely |  |
| SucA | SAOUHSC_01418 | -11.98 | 244 | 299 | not likely |  |
| HemA | SAOUHSC_01776 | -13.58 | 274 | 280 | not likely |  |
| AcsA | SAOUHSC_01846 | -10.51 | -14 | 33 | likely |  |
| HemY | SAOUHSC_01960 | -12.78 | -9 | 36 | likely |  |
|  | SAOUHSC_02003 | -15.12 | -23 | 8 | likely |  |
| RocF | SAOUHSC_02409 | -14.93 | 136 | 156 | not likely |  |
| Rnd2 | SAOUHSC_02525 | -17.59 | 29 | 92 | not likely |  |
| FdhA | SAOUHSC_02582 | -17.75 | -37 | 10 | likely | -37 to +3 |
|  | SAOUHSC_02760 | -16.22 | -52 | -6 | likely | -45 to +11 |
|  | SAOUHSC_02861 | -14.03 | -54 | 9 | likely |  |
| BstA | SAOUHSC_03028 | -18.28 | -38 | 46 | likely |  |

**Interaction energy:** mean of the predicted interaction energies based on the CopraRNA2 predictions (Supplemental Table S2)

**Interaction region:** largest interaction region predicted based on the CopraRNA2 predictions (Supplemental Table S2)

**RBS interaction:** indication if the interaction region overlaps the RBS (-20 to +15 bp relative to start codon)

# Supplementary Figures


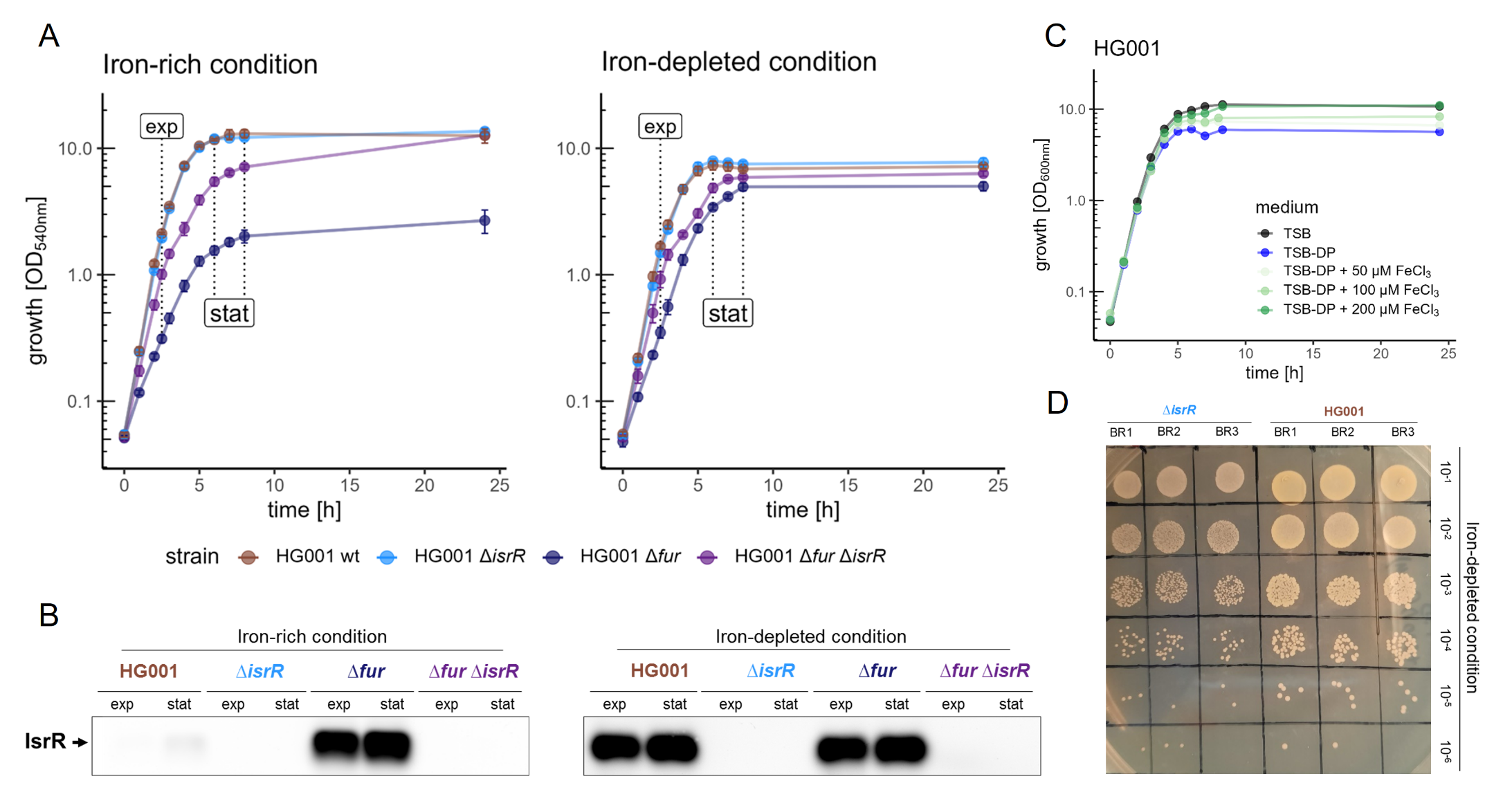


Figure S1. Iron-related growth of *S. aureus* strains and expression of *isrR*. **(A)** Growth curves of *S. aureus* HG001, HG001 ∆*isrR*, HG001 ∆*fur* and HG001 ∆*fur* ∆*isrR* under iron-rich (TSB) and iron-depleted (TSB + 600 µM DP) conditions. Four independent experiments were performed. **(B)** Northern blot analysis of IsrR sRNA. Samples were harvested in exponential (exp) at 2.5 h and stationary growth phase (stat) at 6 h (wild type and ∆*isrR*) or 8 h (∆*fur* and ∆*fur*∆*isrR*). For each RNA sample, 4 µg of total RNA was loaded. **(C)** Growth curves of *S. aureus* HG001 under iron-rich (TSB), iron-depleted (TSB-DP) and added-iron (TSB-DP + FeCl_3_) conditions. Iron repletion of TSB-DP was achieved by addition of FeCl_3_ dissolved in 0.1 M HCl and of 0.1 M NaOH. **(D)** Spot test of HG001 and HG001 Δ*isrR*. Bacterial cells growing exponentially under iron deficiency (TSB-DP) were spotted in dilution series from 10^-1^ to 10^-6^ at an OD_540_ of 1.0 onto a TSB agar plate containing 600 µM dipyridyl (TSB-DP). Three independent tests (BR1 -BR3) are presented for each strain and the plate was incubated at 37 °C overnight.

**
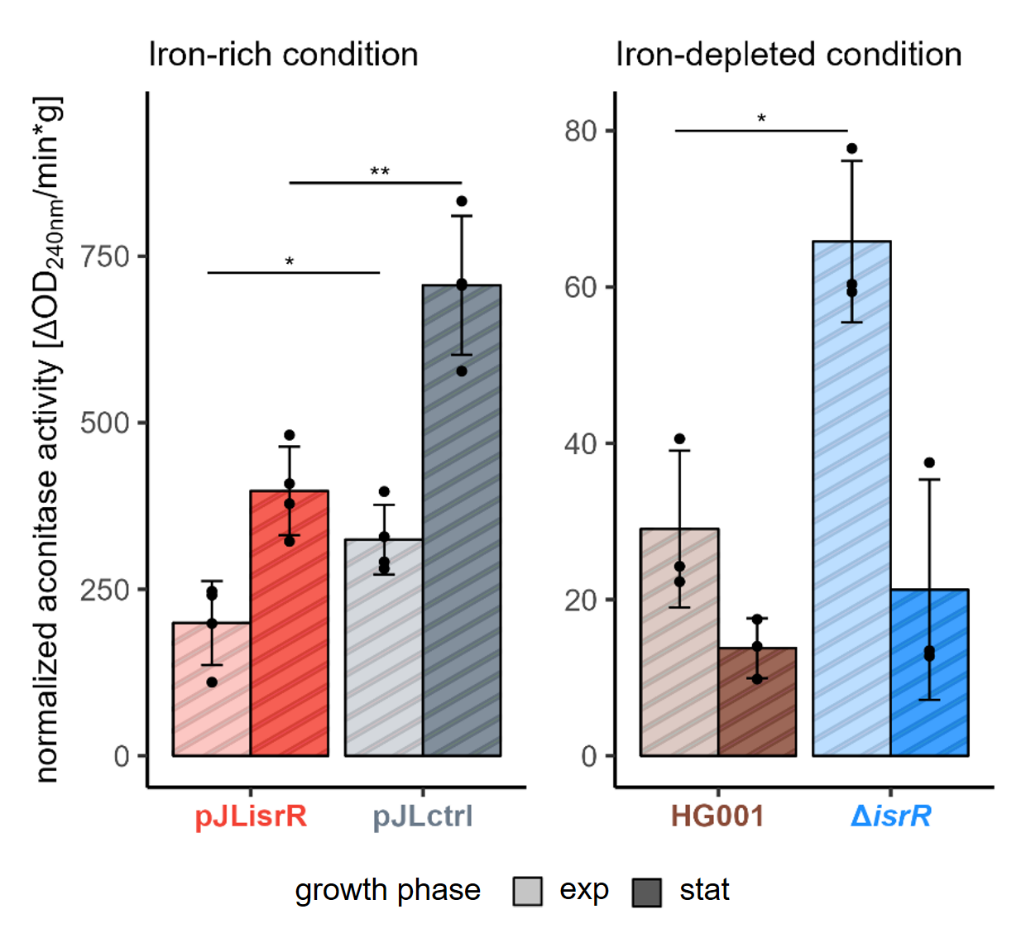
**

Figure S2. Aconitase activity determined as absorbance units per minute and normalized to the respective protein concentration. The indicated strains were grown in TSB or in TSB-DP. The bars depict the mean enzyme activity measured in duplicate for two biological replicates for the iron-rich condition and triplicates for the iron-poor condition. Error bars represent the standard deviation. Statistics: Welch-t-test (p ≤ 0.001: ***, p ≤ 0.01: **, p ≤ 0.05: *). Statistics between the growth phases were not considered.


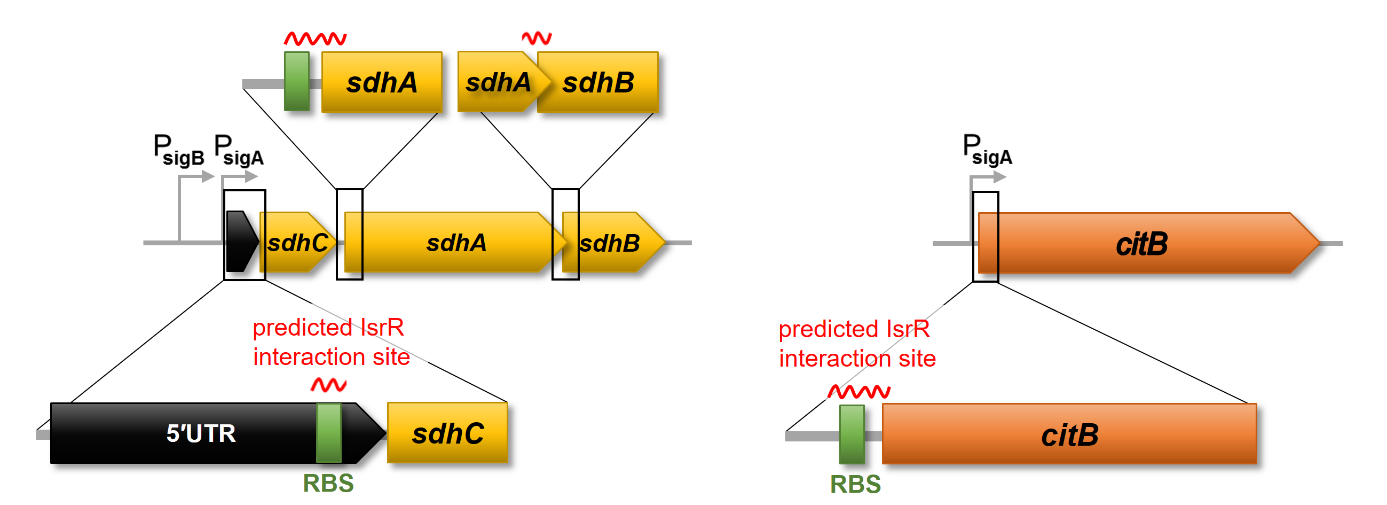


Figure S3. Schematic representation of the *S. aureus* operon organization of *sdhCAB* and *citB* and their predicted interaction region with IsrR.

**
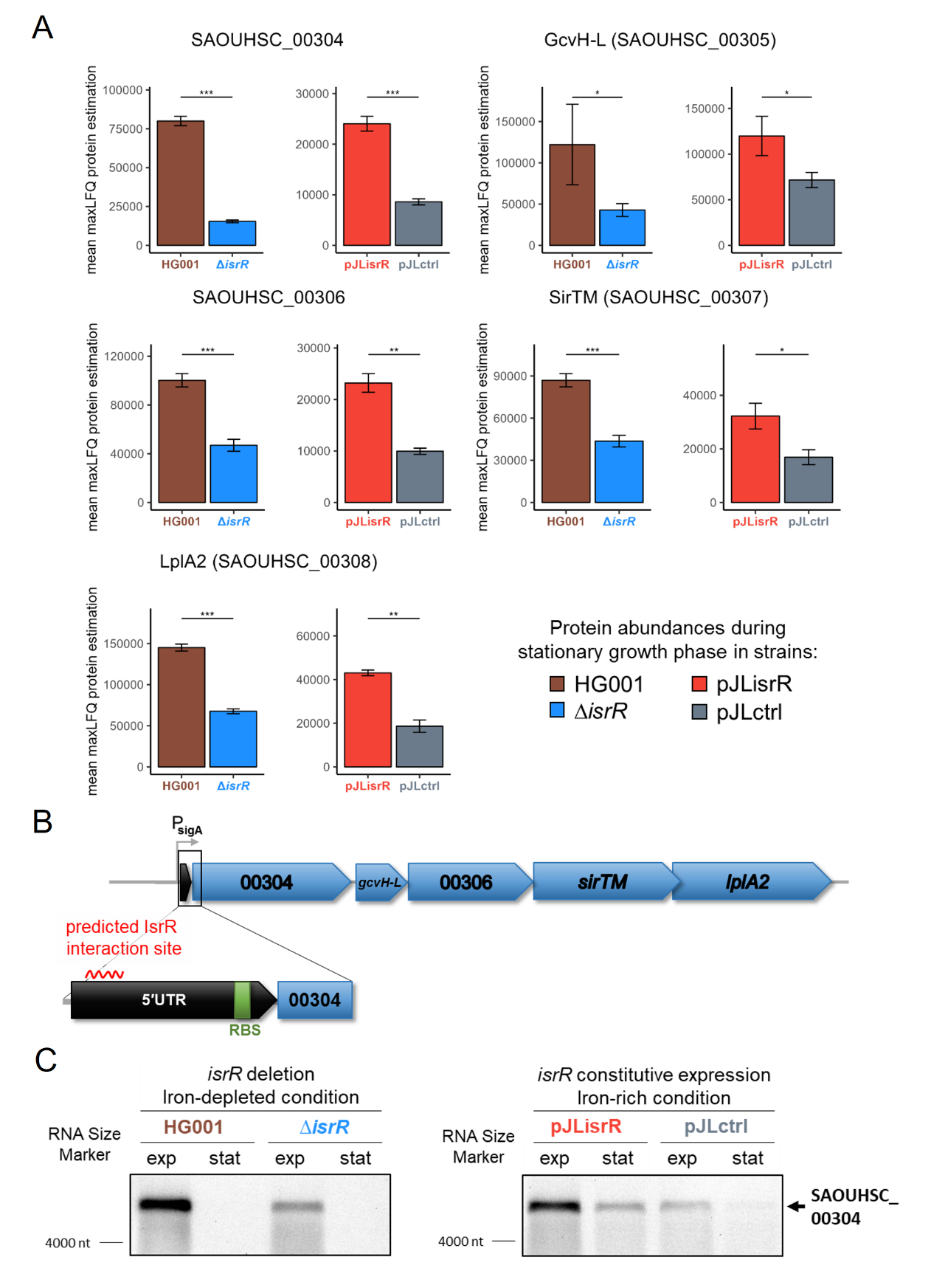
**

Figure S4. Protein levels and regulatory effects of IsrR on the *sirTM* Operon. **(A)** Protein levels of *sirTM* operon. The bar chart depicts the amount (mean maxLFQ protein level) of indicated proteins in stationary (stat) growth phase between IsrR-expressing (HG001, pJLisrR) and non-expressing strains (∆*isrR*, pJLctrl). Error bars represent the standard deviation of the biological replicates. Statistics: Welch-t-test on protein levels (p < 0.001: ***, p < 0.01: **, p < 0.05: *). **(B)** Schematic representation of *sirTM* operon structure and the predicted interaction region with IsrR. **(C)** The effect of IsrR on the mRNA abundance of SAOUHSC_03004 was examined by Northern blot analysis.


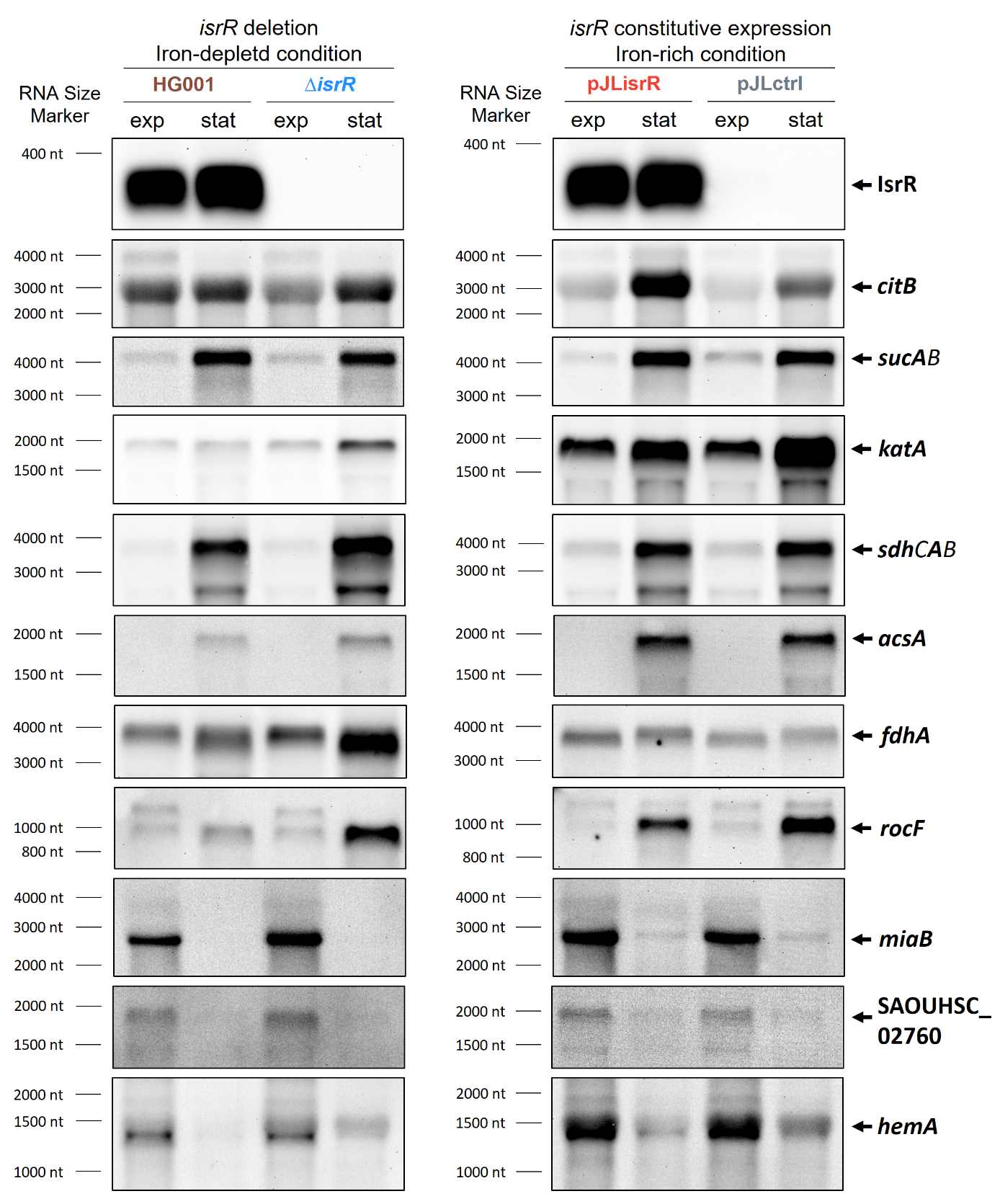


Figure S5. The mRNA levels of ten identified IsrR targets negatively affected by presence of the sRNA were determined by Northern blot analysis. RNA samples were obtained at the same time points and under the same cultivation conditions as the proteome samples (left panel: first experimental approach, right panel: second experimental approach). For each sample 4 µg of total RNA was loaded per lane. Shown are representative results from two biological replicates.


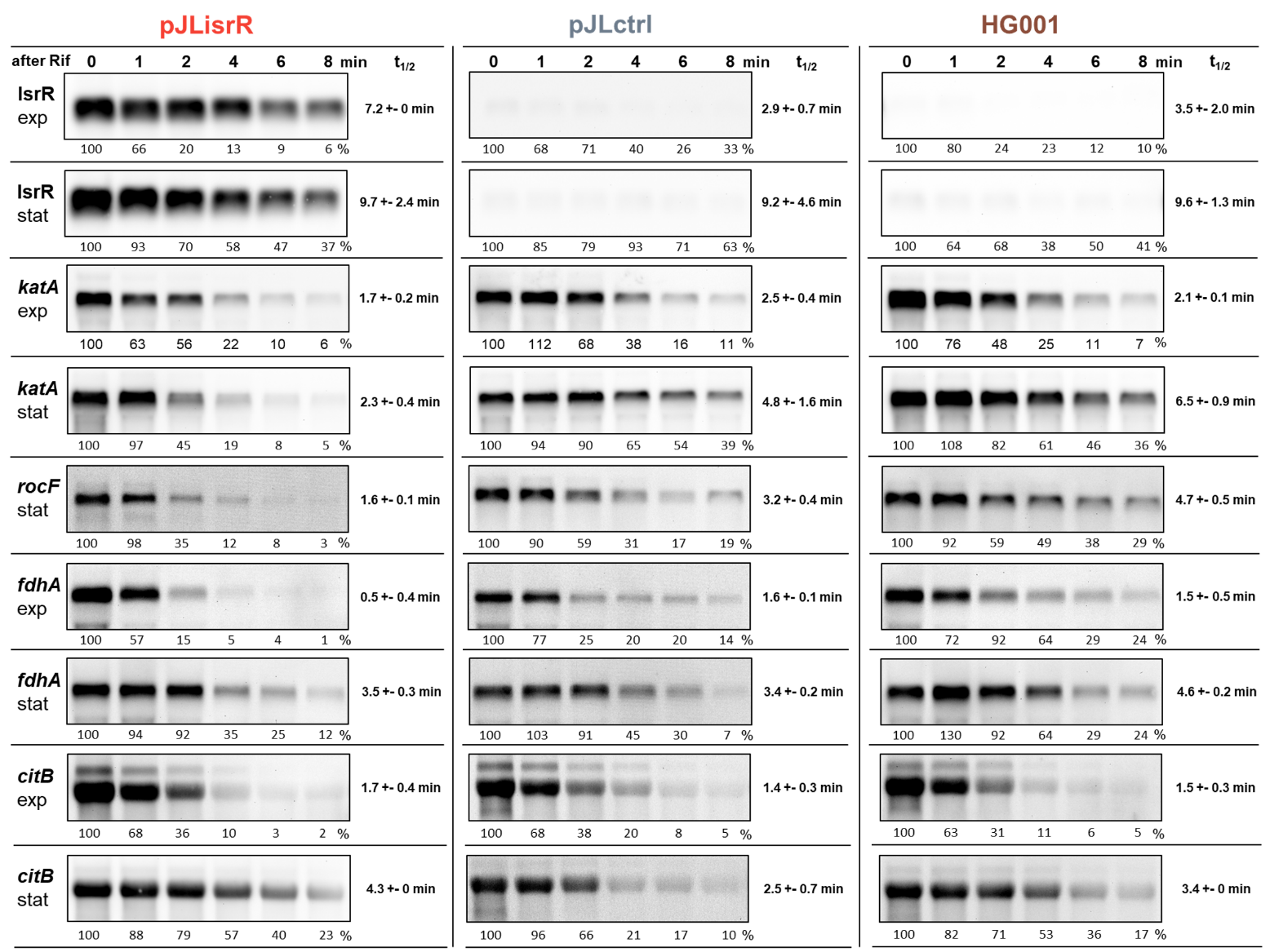


Figure S6. Level and stability of selected experimentally identified mRNAs from IsrR targets. RNA samples were obtained at exponential (exp) and stationary (stat) growth phase and under iron-rich condition (TSB). For each sample 4 µg of total RNA was loaded per lane. Half-life comparison of specific mRNAs in the pJLisrR (left), pJLctrl (middle) and wild-type (right) strain. The half-lives were determined in two independent experiments and one representative result from each is shown.


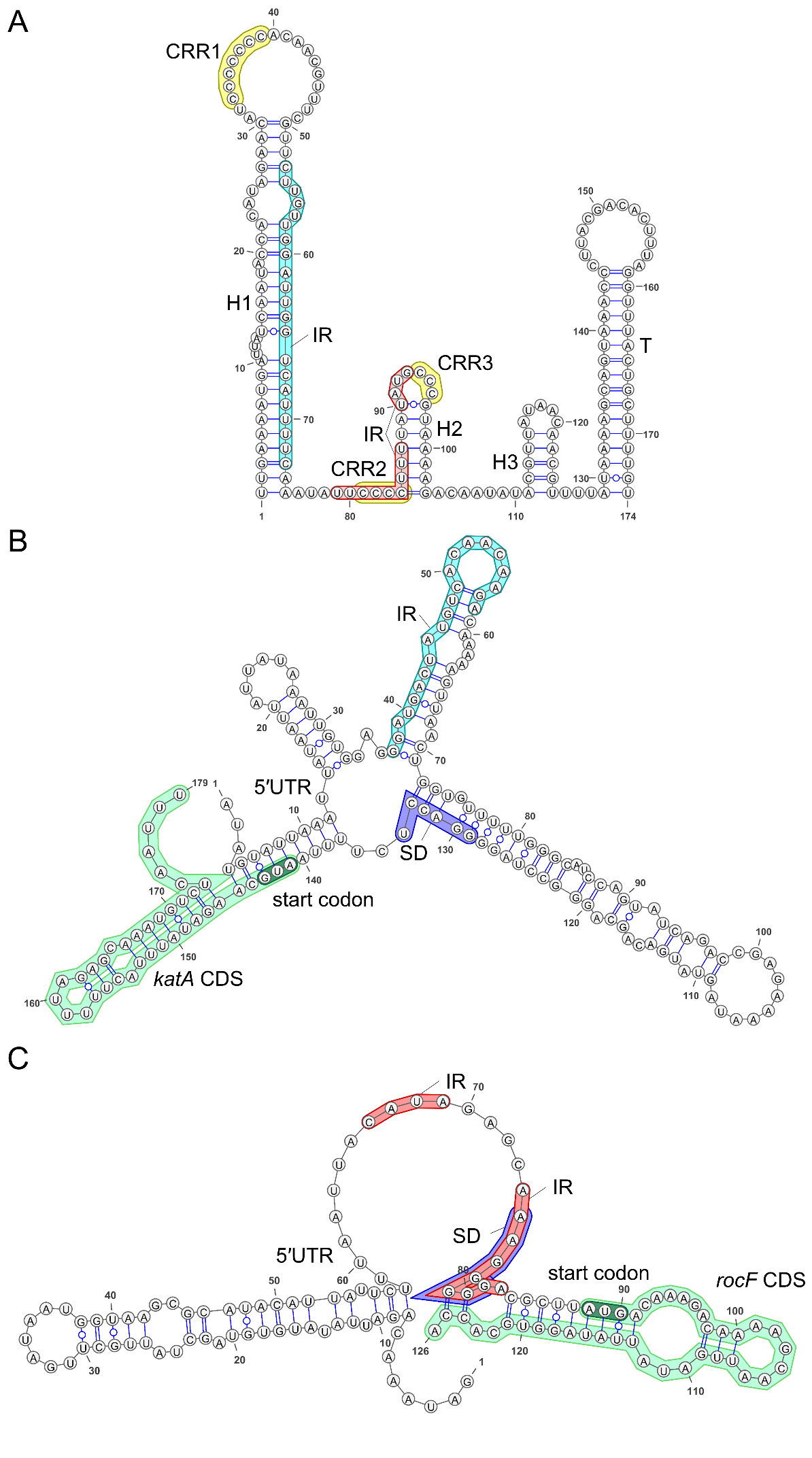


Figure S7. Secondary structure models **(A)** The IsrR secondary structure model according to Coronel-Tellez et al. (2022). The following regions of IsrR are indicated: stem-loop structures H1 to H3, a rho-independent transcription terminator (T), three C-rich regions (CRR1 to CRR3, yellow), the predicted *katA* interaction region (IR, light blue) according to CopraRNA2 (Wright et al. 2013), and the predicted *rocF* interaction region (IR, red) according to IntaRNA 2.0 (Mann et al. 2017). **(B)** Secondary structure models of the 5′UTR (S550) and *katA* mRNA (13 codons) and **(C)** of the 5′UTR and *rocF* mRNA (13 codons) beginning from the transcription start according to Mäder et al. (2016). Both models were obtained with ViennaRNA Web Services (Gruber et al. 2008), the results were calculated with RNAfold 2.6.3. The boxes surrounding the sequences indicate the following regions: light blue: predicted *katA*-IsrR interaction region (IR) according to CopraRNA2 (Wright et al. 2013), red: predicted *rocF*-IsrR interaction region (IR) according to IntaRNA 2.0 (Mann et al. 2017), dark blue: Shine-Dalgarno (SD) sequence, dark green: start codon, light green: *katA*/*rocF* coding DNA sequence (CDS). The secondary structure models were visualized using the Visualization Applet for RNA (VARNA), utilizing the Radiate (A) and the Nucleic Acid View (B-C) algorithms (Darty et al. 2009).

4 References

Bae, Taeok; Schneewind, Olaf (2006): Allelic replacement in Staphylococcus aureus with inducible counter-selection. In: *Plasmid* 55 (1), S. 58–63. DOI: 10.1016/j.plasmid.2005.05.005.

Bolivar, F.; Rodriguez, R. L.; Greene, P. J.; Betlach, M. C.; Heynker, H. L.; Boyer, H. W. et al. (1992): Construction and characterization of new cloning vehicles. II. A multipurpose cloning system. 1977. In: *Biotechnology (Reading, Mass.)* 24, S. 153–171.

Brückner, R. (1997): Gene replacement in Staphylococcus carnosus and Staphylococcus xylosus. In: *FEMS microbiology letters* 151 (1), S. 1–8. DOI: 10.1111/j.1574-6968.1997.tb10387.x.

Coronel-Tellez, Rodrigo H.; Pospiech, Mateusz; Barrault, Maxime; Liu, Wenfeng; Bordeau, Valérie; Vasnier, Christelle et al. (2022): sRNA-controlled iron sparing response in Staphylococci. In: *Nucleic acids research* 50 (15), S. 8529–8546. DOI: 10.1093/nar/gkac648.

Darty, Kévin; Denise, Alain; Ponty, Yann (2009): VARNA: Interactive drawing and editing of the RNA secondary structure. In: *Bioinformatics (Oxford, England)* 25 (15), S. 1974–1975. DOI: 10.1093/bioinformatics/btp250.

Geiger, Tobias; Francois, Patrice; Liebeke, Manuel; Fraunholz, Martin; Goerke, Christiane; Krismer, Bernhard et al. (2012): The stringent response of Staphylococcus aureus and its impact on survival after phagocytosis through the induction of intracellular PSMs expression. In: *PLoS pathogens* 8 (11), e1003016. DOI: 10.1371/journal.ppat.1003016.

Gruber, Andreas R.; Lorenz, Ronny; Bernhart, Stephan H.; Neuböck, Richard; Hofacker, Ivo L. (2008): The Vienna RNA websuite. In: *Nucleic acids research* 36 (Web Server issue), W70-4. DOI: 10.1093/nar/gkn188.

Harrell Jr, Frank E. (2022): Hmisc: Harrell Miscellaneous. Version R package version 4.7-1. Online verfügbar unter https://CRAN.R-project.org/package=Hmisc.

Hester, Jim; Wickham, Hadley; Bryan, Jennifer (2021): vroom: Read and Write Rectangular Text Data Quickly. Version R package version 1.5.7. Online verfügbar unter https://CRAN.R-project.org/package=vroom.

Kassambara, Alboukadel (2020): ggpubr: 'ggplot2' Based Publication Ready Plots. Version R package version 0.4.0. Online verfügbar unter https://CRAN.R-project.org/package=ggpubr.

Kassambara, Alboukadel (2021): rstatix: Pipe-Friendly Framework for Basic Statistical Tests. Version R package version 0.7.0. Online verfügbar unter https://CRAN.R-project.org/package=rstatix.

Lê, Sébastien; Josse, Julie; Husson, François (2008): FactoMineR : An R Package for Multivariate Analysis. In: *J. Stat. Soft.* 25 (1). DOI: 10.18637/jss.v025.i01.

Li, Mamie Z.; Elledge, Stephen J. (2012): SLIC: a method for sequence- and ligation-independent cloning. In: *Methods in molecular biology (Clifton, N.J.)* 852, S. 51–59. DOI: 10.1007/978-1-61779-564-0_5.

Mäder, Ulrike; Nicolas, Pierre; Depke, Maren; Pané-Farré, Jan; Debarbouille, Michel; van der Kooi-Pol, Magdalena M. et al. (2016): Staphylococcus aureus Transcriptome Architecture: From Laboratory to Infection-Mimicking Conditions. In: *PLoS genetics* 12 (4), e1005962. DOI: 10.1371/journal.pgen.1005962.

Mann, Martin; Wright, Patrick R.; Backofen, Rolf (2017): IntaRNA 2.0: enhanced and customizable prediction of RNA-RNA interactions. In: *Nucleic acids research* 45 (W1), W435-W439. DOI: 10.1093/nar/gkx279.

Mike FC; L Davis, Trevor; Wickham, Hadley (2022): ggpattern: 'ggplot2'. Version R package version 1.0.1. Online verfügbar unter https://CRAN.R-project.org/package=ggpattern.

Pedersen, Thomas Lin (2020): patchwork: The Composer of Plots. Version R package version 1.1.1. Online verfügbar unter https://CRAN.R-project.org/package=patchwork.

Pham, Thang V.; Henneman, Alex A.; Jimenez, Connie R. (2020): iq: an R package to estimate relative protein abundances from ion quantification in DIA-MS-based proteomics. In: *Bioinformatics (Oxford, England)* 36 (8), S. 2611–2613. DOI: 10.1093/bioinformatics/btz961.

Schauberger, Philipp; Walker, Alexander (2021): openxlsx: Read, Write and Edit xlsx Files. Version R package version 4.2.5. Online verfügbar unter https://CRAN.R-project.org/package=openxlsx.

Slowikowski, Kamil (2021): ggrepel: Automatically Position Non-Overlapping Text Labels with 'ggplot2'. Version R package version 0.9.1. Online verfügbar unter https://CRAN.R-project.org/package=ggrepel.

Suomi, Tomi; Hiissa, Jukka; Elo, Laura L. (2021): PECA: Probe-level Expression Change Averaging. Version R package version 1.30.0.

Thakker-Varia, S.; Ranzini, A. C.; Dubin, D. T. (1985): Ribosomal RNA methylation in Staphylococcus aureus and Escherichia coli: effect of the "MLS" (erythromycin resistance) methylase. In: *Plasmid* 14 (2), S. 152–161. DOI: 10.1016/0147-619x(85)90075-7.

Wickham, Hadley; Averick, Mara; Bryan, Jennifer; Chang, Winston; McGowan, Lucy; François, Romain et al. (2019): Welcome to the Tidyverse. In: *JOSS* 4 (43), S. 1686. DOI: 10.21105/joss.01686.

Wickham, Hadley; Bryan, Jennifer (2022): readxl: Read Excel Files. Version R package version 1.4.0. Online verfügbar unter https://CRAN.R-project.org/package=readxl.

Wickham, Hadley; Hester, Jim; Bryan, Jennifer (2022): readr: Read Rectangular Text Data. Version R package version 2.1.2. Online verfügbar unter https://CRAN.R-project.org/package=readr.

Wickham, Hadley; Seidel, Dana (2022): scales: Scale Functions for Visualization. Version R package version 1.2.0. Online verfügbar unter https://CRAN.R-project.org/package=scales.

Wilke, Claus O.; Wiernik, Brenton M.: ggtext: Improved Text Rendering Support for 'ggplot2'. Version R package version 0.1.2. Online verfügbar unter https://CRAN.R-project.org/package=ggtext.

Wright, Patrick R.; Richter, Andreas S.; Papenfort, Kai; Mann, Martin; Vogel, Jörg; Hess, Wolfgang R. et al. (2013): Comparative genomics boosts target prediction for bacterial small RNAs. In: *Proceedings of the National Academy of Sciences of the United States of America* 110 (37), E3487-96. DOI: 10.1073/pnas.1303248110.
